# Problematic smartphone use and specific problematic Internet uses among university students and associated predictive factors: a systematic review

**DOI:** 10.1007/s10639-022-11437-2

**Published:** 2022-11-29

**Authors:** Magdalena Sánchez-Fernández, Mercedes Borda-Mas

**Affiliations:** Department of Personality, Assessment and Psychological Treatment, University of Seville (Universidad de Sevilla), C. Camilo José Cela, S/N, 41018 Seville, Spain

**Keywords:** Problematic smartphone use, Problematic social media use, Internet gaming disorder, Predictors, Psychological variables, College students

## Abstract

University students are a high-risk population with problematic online behaviours that include generalized problematic Internet/smartphone use and specific problematic Internet uses (for example, social media or gaming). The study of their predictive factors is needed in order to develop preventative strategies. This systematic review aims to understand the current state of play by examining the terminology, assessment instruments, prevalence, and predictive factors associated with problematic smartphone use and specific problematic Internet uses in university students. A literature review was conducted according to the PRISMA guidelines using four major databases. A total of 117 studies were included, divided into four groups according to the domain of problem behaviour: problematic smartphone use (*n* = 67), problematic social media use (*n* = 39), Internet gaming disorder (*n* = 9), and problematic online pornography use (*n* = 2). Variability was found in terminology, assessment tools, and prevalence rates in the four groups. Ten predictors of problematic smartphone use, five predictors of problematic social media use, and one predictor of problematic online gaming were identified. Negative affectivity is found to be a common predictor for all three groups, while social media use, psychological well-being, and Fear of Missing Out are common to problematic smartphone and social media use. Our findings reaffirm the need to reach consistent diagnostic criteria in cyber addictions and allow us to make progress in the investigation of their predictive factors, thus allowing formulation of preventive strategies.

## Introduction

In recent years, the global percentage of Internet users has grown exponentially and smartphone has become the main device in its access (We Are Social and Hootsuite, 2022). The expansion of Internet access has changed the way people live, work, communicate, and learn and has become an essential environment in their development (Pinho et al., 2021). In the education sector, incorporation of the use of Internet services has led to multiple improvements in the teaching–learning process (Wu et al., 2010), and specifically at universities, has allowed eliminating geographical barriers and increasing flexibility (Santhanam et al., 2008; Yakubu et al., 2020).

However, university students do not only use the Internet for educational and academic purposes, but also to look for information, random navigation, entertainment, communication, gaming, social networks, and online shopping and, to a lesser extent, gambling and obtaining sexual information (Adorjan et al., 2021; Anand et al., 2018; Balhara et al., 2019; Maqableh et al., 2021; Servidio, 2014; Zenebe et al., 2021). These coincide with the purposes of smartphone use by this population (Coban & Gundogmus, 2019; Matar Boumosleh & Jaalouk, 2017).

The widespread availability of the Internet through smartphones and other devices is associated with multiple benefits, such as access to information and a space for social communication and entertainment. (e.g., Maia et al., 2020; Manago et al., 2012). However, Internet penetration in everyday life is a serious problem for an increasing number of people, rising to the level of problematic Internet use (PIU) or problematic smartphone use (PSU). These problematic behaviours are associated with negative consequences such as poor academic performance (Anderson et al., 2017; Grant et al., 2019), psychological distress (Busch & McCarthy, 2021; Chen et al., 2020; Odacı & Çikrikci, 2017; Radeef & Faisal, 2018; Weinstein et al., 2015), and disturbed sleep and daytime sleepiness (Ferreira et al., 2017; Yang et al., 2020a), to name a few.

### Problematic Internet and smartphone use

Although there was already concern about addictive use of the internet by the end of the last century (Griffiths, 1995; Young, 1998a), today, there is currently greater recognition of technological addictions in mental health by both the American Psychiatric Association (APA, 2013) and the World Health Organization (WHO, 2018), and excessive use of digital technologies has been recognised as a public health issue (WHO, 2015).

Today, several terms are often used to describe the phenomenon, such as addiction (Young, 1998a) or internet dependency (Dowling & Quirk, 2009). Among these, the term “problematic Internet use” (hereinafter, PIU) stands out, and it is defined as a pattern of maladaptive Internet use characterised by loss of control, the appearance of negative consequences, and obsessive thoughts when the Internet cannot be accessed (D'Hondt et al., 2015). This is an umbrella term (Fineberg et al., 2018) and accommodates the broad spectrum of non-adaptive behaviours online, which go beyond behavioural addiction (Billieux et al., 2015b; Starcevic, 2013). However, the terms "Internet addiction" and PIU are used inconsistently in the literature (Sánchez-Fernández et al., 2022).

Smartphone, because it is portable and gives easy access to internet, has the potential to create high dependency and is a powerful risk factor for problematic and addictive behaviours (Aljomaa et al., 2016; Carbonell et al., 2018). As a result, problematic smartphone use (PSU) is now being discussed more and more, such as excessive smartphone use that interferes with various areas of a person's life (Billieux et al., 2015a).

The current debate is whether PSU can be considered a sub-category of PIU or whether it is an independent phenomenon (Cheever et al., 2018). Recent studies have found that PIU and PSU overlap in some, but not all key features (Lee et al., 2020; Tateno et al., 2019), while others have established that these problematic behaviours all overlap (Kittinger et al., 2012; Montag et al., 2015).

Recent epidemiological studies have found a large variability in the prevalence rates of PIU and PSU in the general population (López-Fernández & Kuss, 2020; Sohn et al., 2019). In the case of university students, variability has also been found in the prevalence rates of PIU (4—51%), which may be explained by the lack of diagnostic criteria and cultural differences between samples (Sánchez-Fernández et al., 2022). However, despite this variability, these problems increase over time (López-Fernández & Kuss, 2020; Kuss et al., 2021; Pan et al., 2020; Shao et al., 2018) and university students tend to be at higher risk of PIU (Anderson et al., 2017; Ferrante & Venuleo, 2021; Kuss et al., 2014) and PSU (Roig-Vila et al., 2020).

### Specific problematic Internet uses

PIU/PSU is a broad term that may include a variety of problematic behaviours. In fact, individuals who use the Internet/smartphone excessively do not become addicted to the Internet/smartphone environment but to the behaviours they engage in when they are online (Király & Demetrovics, 2021; Meerkerk et al., 2009). That is why some authors are sceptical about the viability of PIU/PSU as a construct, and favour the examination of specific activities such as playing games or sexual activity (e.g., Starcevic & Aboujaoude, 2017). At the beginning of the century, Davis (2001) made a distinction between two different forms of pathological use of the Internet: general and specific. The general one includes a broader set of behaviours while the specific one refers to engagement with specific Internet functions or applications. Years later, Billieux (2012) argued for the existence of a spectrum of cyber addictions that would include problematic behaviours related to the smartphone, in general, and specific online activities such as video games and online gambling, pornography, and social networks. More recently, some authors have conceptualised problem behaviours mediated by the Internet and smartphones as being within a spectrum of related conditions associated with both shared and unique characteristics (Baggio et al., 2018). In this study, "specific problematic Internet uses" will refer to those problematic online behaviours that can be carried out via smartphone or any other device.

Previous reviews (Kuss et al., 2021; Lopez-Fernandez & Kuss, 2020) have established four main themes in terms of specific PIU: problematic social media use (PSMU), Internet gaming disorder (IGD), problematic Internet pornography use (PIPU), and problematic Internet gambling. These studies show great variability in terms of terminology (addiction coexisting with problematic use, disorder, or dependence, among others), as well as in measurement instruments and prevalence rates. So far, no systematised data focusing on the university student population have been found.

### Conceptualisation of problematic Internet use behaviours

A number of diverse etiological models have been proposed in the conceptualisation of these problem behaviours (Ferrante & Venuleo, 2021). Davis (2001), in his cognitive behavioural model of pathological Internet use, proposes that psychopathology (distal cause) would give rise to the PIU, generalized or specific, through maladaptive cognitions (proximal cause such as low self-efficacy or negative self-evaluation). The behavioural symptoms of PIU are reinforcements of the maladaptive cognitions that result in a vicious circle and maintain pathological behaviour.

On the other hand, the person-affect-cognition-execution (I-PACE) model (Brand et al., 2016, 2019) argues that specific problematic uses of the Internet are the consequence of interactions between predisposing (personality-related characteristics, social cognitions, biopsychological factors and motivation to use), moderating (coping styles and Internet-related cognitive biases) and mediating (affective and cognitive responses to situational triggers) factors in combination with reduced executive functioning. These associations would be maintained by Pavlovian and instrumental conditioning processes within an addiction process. The authors also assume that the medium (internet, smartphone) is secondary in the origin of these problem behaviours and that, among the psychological and neurobiological mechanisms, some are common and others specific to each addictive behaviour (such as specific personality profiles) (Brand et al., 2016, 2019).

### Risk and protective factors for problematic internet usage behaviours

Research on shared risk and protective factors unique to the spectrum of online problem behaviours is essential for advancing their conceptualisation and prevention, which in turn will have clear implications for the overall health and well-being of university students (Tugtekin et al., 2020). Problem behaviours mediated by the Internet and smartphones are associated with both shared and unique risk factors (Baggio et al., 2018; Billieux, 2012).

Previous reviews have examined risk factors for PIU, finding that being younger, being male, a higher family socio-economic status, duration of use, social networking and gaming, neuroticism, impulsivity, loneliness, depression, anxiety and general psychopathology increase the risk of generalized PIU (Aznar Díaz et al., 2020; Kuss et al, 2014, 2021); depression and aggression were the main risk factors for online gaming addiction, Internet gambling problems were associated with lower emotional intelligence and psychological distress, and problematic online pornography use was most frequently related to relationship problems, disruptive worry and behavioural dysregulation (Kuss et al., 2021). On the other hand, with regard to PSU, the review by Wacks and Weinstein (2021) concludes that it is associated with psychiatric, cognitive, emotional, medical and brain alterations. For their part, Busch and McCarthy's (2021) review of the predisposing factors to PSU found a great variety of backgrounds divided into four categories: Control (e.g. self-control or tolerance of uncertainty), Emotional health (e.g. anxiety and depression), Physical health (e.g. Individual's health status), Preconditions (e.g. family characteristics), Professional performance (e.g. academic performance), Social performance (e.g. personality) and Technology features (e.g. type of mobile phone use).

However, no consistent findings have been found in research on predictors of generalized and specific PIU and PSU in the university student population.

Prior to this study, the authors reviewed studies on risk and protective factors for generalized PIU in university students. Ten predictive factors for PIU have been identified and divided into three categories (patterns of use, psychological variables, and lifestyles). Among these, nine were risk factors (time spent online, video games, depression, negative affect, life stress, maladaptive cognitions, impulsivity, poor sleep quality, substance use (alcohol and drugs), and one was a protective factor (conscientiousness). However, all studies that focus on other technologies-related problem behaviours, such as PSU or specific PIU, were excluded from this review.

### The purpose of the study

Consequently, the aim of this systematic review is to examine the studies of predictors of PSU and specific PIU (online gaming, social networking, online gambling, and online pornography) in university students that have been published since the inclusion of IGD in the DSM-5.

The research questions are: 1. What terminology is used to refer to PSU and specific PIU?, 2. What are the assessment tools used in the PSU and specific PIU evaluation?, 3. What is the prevalence of PSU and specific PIU?, 4. What are the risk and protective factors associated with PSU and specific PIU? From these questions, the objectives are as follows: 1. To become familiar with the terminology used, 2. To review the assessment tools, 3. To analyse prevalence and 4. To study the risk and protective factors associated with PSU and specific PIU.

## Methodology

Systematic review methodology was used (Page et al., 2021). We included scientific research articles published between 2013, the year when "Internet gaming disorder" (IGD) in DSM-5 (APA, 2013) was officially recognised, and the expansion of smartphone use (Carbonell et al., 2018), and 2021, both years included, on predictive, risk and protective factors associated with PSU and specific problematic internet use (e.g., social networking) in university students. The Web of Science, Proquest (PsycINFO and Medline) and Scopus databases were used between October and December 2021. The keyword strategy used the terms, clusters, and Boolean operators listed in Table 1 (also translated into Spanish, French, and Italian). The search was done by article title, abstract and keywords.

**Table 1:** Search strategy

Identifier 1	Identifier 2	Identifier 3	Identifier 4
(Problem* OR dependen* OR excess* OR compuls* OR addict* OR patholog* OR disorder)	(Internet OR smartphone OR “mobile phone” OR “cell* phone” OR “video gam*” OR “online gam*” OR “social network*” OR "social media" OR “online pornography”)	(factor* OR variabl* OR caus* OR antecedent* OR predictor*)	(undergraduate* OR "university student*" OR "college student*")

### Inclusion and exclusion criteria

The inclusion criteria were: (1) scientific articles; (2) study factors predicting PSU or specific PIU through predictive modelling; (3) university students from more than one area of knowledge; (4) 17 years or older; (5) use of validated instrument; (6) quantitative empirical data; (7) reported effect size; (8) access to full text; and (9) written in English, Spanish, French and Italian.

The exclusion criteria were: (1) studying predictors of PIU or other behavioural addictions that do not involve Internet use (e.g. offline gambling) or substance-related addictions (e.g. alcoholism); (2) lack of relevant data; (3) non-university sample; (4) under 17 years of age; (5) university students from a single field of knowledge; (6) sample collected during the covid-19 lockdown; (7) no predictive statistical model; (8) no use of validated instruments; (9) validation studies of assessment instruments; (10) unreported effect size; and (12) sources other than peer-reviewed journals (e.g. non-peer-reviewed journals, conference abstracts, chapters, books, corrections).

### Selection of articles

**Fig. 1:**
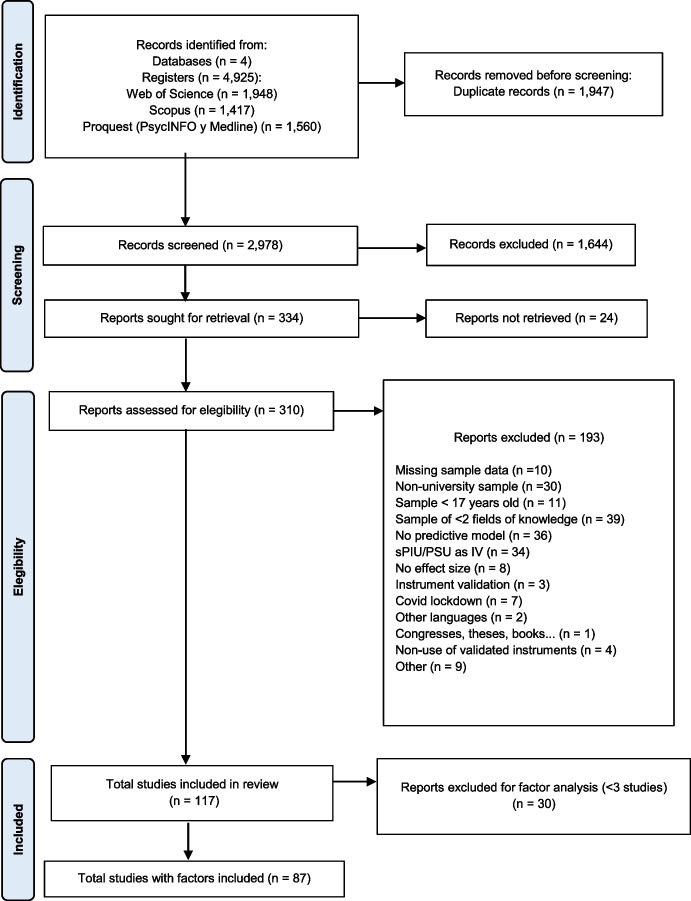
PRISMA Diagram of study selection processes

The PRISMA protocol guidelines were followed (Page et al., 2021) (See Fig. 1). Our initial sample contained 117 articles. They were included in the present review after screening duplicates and articles that met the inclusion criteria mentioned, but not the exclusion criteria. The included studies were organized into four main subjects, one of them referring to a generalized problematic use of smartphones: Problematic Smartphone Use (PSU) and three on specific problematic uses of the internet: Problematic Social Media Use (PSMU), Internet Gaming Disorder (IGD) and Problematic Internet Pornographic Use (PIPU). Since they analysed more than one variable, some studies have been repeated in two subject groups.

At a second stage, articles whose predictive factors were supported by at least 3 studies were chosen, in order to address Objective 4. As a result of this second analysis, 83 articles were selected that studied 10 PSU factors, 5 PSMU factors, and 1 IGD factor.

### Data extraction

The characteristics of the 117 studies selected are shown in Table 2. In terms of effect size, betas (ß), odds ratio (OR) and coefficient of determination (R2) were included. For betas, the cut-off points are used: Very small > 0 to < 0.1, small ≥ 0.1 to < 0.3, medium ≥ 0.3 to < 0.5 and large ≥ 0.5 (Cohen, 2013, Ferguson, 2016). For odds ratios (OR): Very small > 0 to < 1.5, small ≥ 1.5 to < 2, medium ≥ 2 to < 3, and large ≥ 3 (Sullivan & Feinn, 2012). With respect to the coefficient of determination (R2): Very small < 0.02, small ≥ 0.02, medium ≥ 0.13 and large ≥ 0.26 (Dominguez-Lara, 2017).

**Table 2:** Descriptive characteristics of selected articles in alphabetical order (*N* = 117)

	Author, year of publication	Country	Sample size (proportion of female)	Age sample (years: range, M (SD))	Variable	Assessment tool^a^ (prevalence: M (SD)/% (cut-off point))	Predictive factors	Stadistical model^b^ (number of predictors), Measure of association	Direction and effect size^c^	QA^d^
Problematic smartphone use (PSU)										
Prospective cohort studies										
1	Cui et al., 2021	China	1181 (51%)	18 – 21, 18.91 (0.85)	Problematic mobile phone use	MPATS (37.08 (13.62))	(I) T1 PMPU (II) T1 Bedtime procrastination (III) T1 Depressive symptoms	CL (adjusting gender and age, bedtime procrastination, sleep quality and depressive symptoms) (I) 0.423** (II) 0.092** (III) 0.151**	(I) M ( +) (II) VS ( +) (III) S ( +)	Good (14)
2	Elhai et al., 2018b	USA	261 (76.9%)	19.73 (0.52)	Problematic smartphone use	SAS-SV (26.31 (10.35))	(I) Distress tolerance (II) Midfulness (III) Smartphone use frequency	SEM (Sex, age, depression, anxiety sensitivity, distress tolerance, mindfulness, smartphone use frecuency) (I) − 0.20* (II) − 0.39*** (III) 0.14*	(I) S (-) (II) M (-) (III) S ( +)	Fair (7, 14)
3	Rozgonjuk et al., 2018	Estonia	366 (79%)	19 – 55, 25.75 (7.70)	Problematic smartphone use	E-SAPS18 (33.58 (12.12))	(I) Social media use in lectures (II) Age	SEM (Trait procrastanation, Social Media Use in Lectures, Age, Gender) (I) 0.345*** (II) -0.411***	(I) M ( +) (II) M (-)	Fair (2, 7, 14)
4	Yuan et al., 2021	China	341 (75.7%)	21.24 (2.72)	Problematic Smartphone Use	SAS-SV (35.23 (10.58))	(I) Gender (being female) (II) Depression symptoms (III) FoMO (IV) IGD (IGD)	SEM (Age, gender, fear of missing out, IGD, depression) (I) 0.19*** (II) 0.15** (III) 0.18*** (IV) 0.26***	(I) S ( +) (II) S ( +) (III) S ( +) (IV) S ( +)	Fair (3, 13, 14)
5	Zhang et al., 2021	China	352 (55%)	17 – 23, 19.30 (1.16)	Mobile Phone addiction	MPATS (2.60 (0.60))	(I) Boredom proneness W1 (II) Mobile phone addiction W1	CL (boredom proneness (W1, W2), mobile phone addiction (W1)) (I) 0.10* (II) 0.69***	(I) S ( +) (II) L ( +)	Good (14)
Cross-sectional studies										
6	Abbasi et al., 2021	Malaysia	250 (58%)	80% 18–32	Smartphone addiction	SAS-SV (NR)	(I) Entertainment (II) Social networking sites (SNS) use (III) Game-related use	SEM (smartphone content i.e. study, entertainment, SNS, and game-related usage) (I) 0.146** (II) 0.128* (III) 0.427***	(I) S ( +) (II) S ( +) (III) M ( +)	Fair (2, 3, 14)
7	Alavi et al., 2020	China	1400 (68%)	18 – 35, 25.17 (4.5)	Smartphone addiction	CPDQ (NR)	(I) Sex [female VS Male] (II) Marital Status [Single VS Married] (III) Bipolar disorder (IV) Depression (V) Anxiety (VI) Somatization (VII) Dependent personality disorder (VIII) Compulsive personality disorder	MLogR (Sex, Age, Marital status, avoidant personality disorder, PTSD, Cychlotymia, Panic Disorder, OCD, Anorexia, Bulimia, bipolar disorder, depression, anxiety, somatization, dependent personality disorder, and compulsive personality disorder) (I) 1.2* 1.7–2.8 (II) 1.5* 1.9–2.5 (III) 4.2* 1.27–14.1 (IV) 4.2* 4.6–39.2 (V) 1.2* 3.8–6.4 (VI) 2.8* 6.8–11.9 (VII) 3.1* 1.38–6.92 (VIII) 3.2* 1.4–6.7	(I) VS ( +) (II) S ( +) (III) L ( +) (IV) L ( +) (V) VS ( +) (VI) M ( +) (VII) L ( +) (VIII) L ( +)	Fair (2, 3)
8	Alosaimi et al., 2016	Saudi Arabia	2367 (56%)	50% 20–24	Problematic use of mobile phones	PUMPS (60.8)	(I) Consequences of the use of smartphones (II) Number of hours spent per day using smartphones (III) Year of study (IV) Number of applications used	MR (consequences of smartphone use (negative lifestyle, poor academic achievement), number of hours per day spent using smartphones, years of study, and number of applications used) (I) 0.564*** (II) 0.225*** (III) 0.086*** (IV) 0.046**	(I) L ( +) (II) S ( +) (III) VS ( +) (IV) VS ( +)	Fair (3, 14)
9	Arpaci & Kocadag Unver, 2020	Turkey	320 (66%)	20.36 (2.35)	Smartphone addiction	SPAI (NR)	Women: (I) Neuroticism (II) Agreeableness (III) Conscientiousness Men: (I) Agreeableness	SEM (gender differences in the relationship between “Big Five personality traits” and smartphone addiction) Women: (I) 0.18* (II) − 0.18* (III) − 0.16* Men: (I) − 0.33*	Women: (I) S ( +) (II) S (-) (III) S (-) Men: (I) M (-)	Fair (2, 3, 14)
10	Bian & Leung, 2014	China	414 (62%)	60.1% 23 – 26	Smartphone addiction	MPPUS (48.48 (12.75))	(I) Grade (II) Shyness (III) Loneliness (IV) Information seeking (V) Utility (IV) Fun seeking	R (Age, Gender, Grade, Family monthly income, Psychological attributes, Shyness, Loneliness, Smartphone usage (Information seeking, Utility, Fun seeking, Sociability)) (I) –0.12* (II) 0.20*** (III) 0.21*** (IV) 0.16*** (V) 0.13** (IV) 0.17***	(I) S (-) (II) S ( +) (III) S ( +) (IV) S ( +) (V) S ( +) (IV) S ( +)	Good
11	Canale et al., 2021	Italy	795 (69.8%)	18 – 35, 23.80 (3.02)	Problematic Mobile Phone Use: Addictive mobile phone use; Antisocial mobile phone use; Dangerous mobile phone use	PMPUQ – SV (Addictive mobile phone use: 12.88 (3.03); Antisocial mobile phone use: 9.78 (2.35); Dangerous mobile phone use: 7.94 (2.95)))	Addictive mobile phone use: (I) negative urgency (II) behavioural inhibition (III) primary psychopathy (IV) social anxiety Antisocial mobile phone use: (I) lack of premeditation (II) sensation seeking (III) aggressive traits (IV) primary psychopathy Dangerous mobile phone use: (I) lack of premeditation (II) sensation seeking (III) primary psychopathy	BA (Social anxiety, Neuroticism, Self-esteem, Psychological distress, Behavioural inhibition, Negative urgency, Positive urgency, Lack of premeditation, Aggression, Primary psychopathy, Secondary psychopathy, Sensation seeking, Extraversion, Reward responsiveness, Drive, Fun seeking) Addictive mobile phone use: (I) 0.22* (II) 0.11* (III) 0.06* (IV) 0.02* Antisocial mobile phone use: (I) 0.17* (II) 0.08* (III) 0.02* (IV) 0.07* Dangerous mobile phone use: (I) 0.30* (II) 0.11* (III) 0.11*	Addictive mobile phone use: (I) S ( +) (II) S ( +) (III) VS ( +) (IV) VS ( +) Antisocial mobile phone use: (I) S ( +) (II) VS ( +) (III) VS ( +) (IV) VS ( +) Dangerous mobile phone use: (I) M ( +) (II) S ( +)	Fair (2, 14)
12	Cebi et al., 2019	Turkey	571 (70.2%)	18—22, 19.03 (1.32)	Problematic mobile phone use	PMPUS (59.87 (16.92))	(I) Experiential self-control (II) Reformative self-control	PM (Experiential Self-Control, Reformative SelfControl) (I) -0.375* (II) -0.142*	(I) M (-) (II) S (-)	Fair (2, 14)
13	Choi et al., 2015	Korea	463 (60%)	20.89 (3.09)	Smartphone addiction	SAS (68.46 (24.95))	(I) Internet Addiction (II) Alcohol Use Disorders (III) State–Trait Anxiety Inventory scores (IV) Gender (being female) (V) Depression (VI) Character Strengths Test–temperance scores	MR (Gender, Internet Adiction Test, State–Trait Anxiety Inventory, Trait Version, Character Strengths Test) (I) 0.184*** (II) 0.251*** (III) 0.224*** (IV) 0.293*** (V) − 0.215*** (VI) − 0.143**	(I) S ( +) (II) S ( +) (III) S ( +) (IV) S ( +) (V) S (-) (VI) S (-)	Fair (2, 14)
14	Coban & Gundogmus, 2019	Turkey	1465 (58.8%)	18—65 (21.10 (1.99))	Smartphone addiction	SAS-SV (46.9%, ≥ 31 M, ≥ 33 F)	(I) using the smartphone for “social media use” (II) using the smartphone for “meeting new friends (III) “Use for studying/academic purpose” (IV) “use to follow the news”	LogR (Social media use, Use for studying/academic purpose, Use for playing games, To meet new friends, Use for communication, For entertainment (watching series, movies, clips), To follow the news, For shopping) (I) OR 2.884, 95% CI 2.085 3.099 (II) OR 2.066, 95% CI 1.535 2.783 (III) OR 0.589, 95% CI 0.434 0.797 (IV) OR 0.645, 95% CI 0.485 0.857	(I) M ( +) (II) M ( +) (III) VS (-) (IV) VS (-)	Fair (3, 14)
15	Enez Darcin et al., 2016	Turkey	367 (62%)	19.5 (1.15)	Smartphone addiction	SAS (87.6 (26.45))	(I) Brief Social Phobia Scale	MR (UCLA Loneliness Scale (UCLA-LS), and Brief Social Phobia Scale (BSPS)) (I) 0.303***	(I) M ( +)	Fair (2, 14)
16	De Pasquale et al., 2019	Italy	400 (61%)	20 – 24, 21.59 (1.43)	Problematic Smartphone Use	SAS-SV ( 41.35 (35.95))	(I) Emotional stability	R (Emotional Stability, Extraversion, Agreeableness, Conscientiousness, Openness to Experience) (I) -0.22**	(I) S (-)	Fair (2, 14)
17	Elhai et al., 2018c	USA	298 (76.8%)	19.45 (2.17)	Problematic smartphone use	SAS (93.47 (25.30))	(I) boredom proneness (II) sex (III) smartphone use frequency	SEM (depression, anxiety, boredom proneness, age, gender, smartphone use frecuency) Direct effects: (I) 0.44*** (II) 0.26*** (III) 0.31**	(I) M ( +) (II) S ( +) (III) M ( +)	Good (14)
18	Elhai et al., 2018a	USA	296 (76.7%)	19.44 (2.16)	Problematic smartphone use	SAS (93.53 (25.38))	(I) FOMO (II) Negative Affectivity	SEM (FoMO, negative affectivity (depression, anxiety, stress, proneness to boredom, and rumination)) (I) 0.33* (II) 0.32*	(I) M ( +) (II) M ( +)	Fair (2, 14)
19	Elhai et al., 2020b	China	1034 (65.3%)	19.34 (1.61)	Problematic smartphone use	SAS-SV (34.92 (11.39))	(I) Age (II) Sex (III) FOMO (IV) Smartphone Use Frequency	SEM (age, sex, FOMO, Smartphone Use Frequency (SUF), depression, anxiety) Direct effects: (I) 0.12*** (II) 0.39*** (III) 0.61*** (IV) 0.22**	(I) S ( +) (II) M ( +) (III) L ( +) (IV) S ( +)	Good (14)
20	Elhai et al., 2020a	USA	316 (66.8%)	19.21 (1.74)	Problematic smartphone use	SAS-SV (27.41 (9.41))	(I) FOMO (II) Non-social use (process use)	SEM (Sex, FOMO, social use, process use, depression, anxiety) (I) 0.59*** (II) 0.18*	(I) L ( +) (II) S ( +)	Good (14)
21	Elhai et al., 2020c	China	1097 (81.9%)	19.38 (1.18)	Problematic smartphone use	SAS-SV (37.36 (9.54))	(I) FOMO (II) Sex (female) (III) Anxiety (IV) Depression (V) Age (VI) Rumination	Ridge, lasso and elastic net algorithms (FOMO, sex, age, depression, anxiety, rumination) (I) 0.23 0.33 0.24 (II) 0.12 0.11 0.11 (III) 0.11 0.11 0.11 (IV) 0.07 0.03 0.06 (V) 0.06 0.03. 0.05 (VI) 0.05 0.01 0.04	(I) S ( +) (II) S ( +) (III) S ( +) (IV) VS ( +) (V) VS ( +) (VI) VS ( +)	Good (14)
22	Erdem & Uzun, 2020	Turkey	485 (35.3%)	17–19 years (*n* = 326, 67.22%) and 20–22 years (*n* = 149, 30.72%)	Smartphone addiction	TSAS (78.93 (23.21))	(I) Age (II) 3–6 h smartphone use versus < 3 h smartphone use (III) > 6 h smartphone use versus 3–6 h smartphone use (IV) 3–6 h Internet use versus < 3 h Internet use (V) > 6 h Internet use versus 3–6 h Internet use (VI) Agreeableness (VII) Conscientiousness (VIII) Neuroticism	HMR (Age, gender, amount of daily average smartphone and Internet use, the big five personality traits (extraversion, agreeableness, conscientiousness, neuroticism, and openness to experience)) (I) − 0.11** (II) 0.23*** (III) 0.14* (IV) 0.12* (V) 0.28*** (VI) -0.15*** (VII) − 0.08* (VIII) 0.15***	(I) S (-) (II) S ( +) (III) S ( +) (IV) S ( +) (V) S ( +) (VI) S (-) (VII) S (-) (VIII) S ( +)	Good (14)
23	Forster et al., 2021	USA	1027 (21.68%)	Over 88% 18 -29	Problematic smartphone use	SAS-SV (24.32%; ≥ 32)	Household dysfunction: (I) Students who reported 1–3 household stressors, (II) Students who had ≥ 4 household stressors (III) Age	LogR (Age, sex, race/ethnicity, financial hardship due to COVID-19, depression, social support from friends, Household Dysfunction (HHD)) (I) AOR 1.40, 95% CI 1.02–1.93 (II) AOR 2.03, 95% CI 1.21–3.40 (III) AOR 0.96, 95% CI 0.93–0.99	(I) VS ( +) (II) S ( +) (III) VS ( +)	Good
24	Giordano et al., 2019	Italy	627 (45%)	18 – 36, 22.77 (3.28)	Problematic smartphone use	SPAI (37.07 (10.60))	Females: (I) Selfie-related behavior Males: (I) Selfie-related behavior	SEM (Age, Selfie-related behaviors and narcissism) Females: (I) 0.315* Males: (I) 0.299*	Females: (I) M ( +) Males: (I) S ( +)	Good (14)
25	Gökçearslan et al., 2016	Turkey	598 (71%)	80% 18–21 20% + 22	Smartphone addiction	SAS-SV (20.96 (7.56))	(I) Smartphone usage (II) Self-regulation (III) Cyberloafing	SEM (Smartphone usage, Self-regulation, Cyberloafing, General self-efficacy) (I) 0.54*** (II) − 0.22*** (III) 0.14***	(I) L ( +) (II) S (-) (III) S ( +)	Fair (2, 14)
26	Gündoğmuş et al., 2021	Turkey	935 (54.4%)	18 – 45, 21.89 (3.27)	Smartphone addiction	SAS-SV (48.6%, ≥ 31 M, ≥ 33 F)	(I) gender (II) number of social media (III) Alexithymia	LogR (age, gender, place of residence, monthly income, number of social media and Alexithymia) (I) OR = 1.496, 95% CI 1.117–2.002, *p* = 0.007 (II) OR = 1.221, 95% CI 1.134–1.315, *p* < 0.001 (III) OR = 1.074, 95% CI 1.059–1.090, *p* < 0.001	(I) VS ( +) (II) VS ( +) (III) VS ( +)	Good
27	Handa & Ahuja, 2020	India	240 (45.4%)	18—25 (88,3% 22–25)	Smartphone addiction	18 items adapted from Zhitomirsky-Geffet and Blau (2016) (2.98 (0.58))	(I) FOMO	SEM (FOMO, loneliness) (I) 0.393***	(I) M ( +)	Fair (2, 14)
28	He et al., 2020	China	668 (55%)	20.05 (1.38)	Excessive smartphone use	SAS-C (2.884 (0.566))	(I) Upward social comparison on SNSs (II) Perceived stress	Mediation analysis (Perceived stress, Upward social comparison) (I) 0.184*** (II) 0.182**	(I) S ( +) (II) S ( +)	Fair (2, 14)
29	Hong et al., 2021	China	206 (53.4%)	18—22	Smartphone addiction	Scale modified from the scale of smartphoneaddiction by Hong, Chiu, and Huang (2012) (Range: 11–59, Average: 33.82 (10.23))	(I) Daily time spent on phone calls and texting (II) Relationship with peers (III) Online descriptive social norms (IV) Remote descriptive social norms (V) Co-present descriptive social norms	PM (Interpersonal relationships (‘Relationship with peers’, ‘Parent–child relationship’, ‘Relationship with remote callers’, and ‘Relationships with cyber friends’), social norm (‘Co-present descriptive social norms’, ‘Co-present injunctive social norms’, ‘Remote descriptive social norms’, ‘Remote injunctive social norms’, ‘Online descriptive social norms’, and ‘Online injunctive social norms’) and smartphone use patterns (daily use time, daily time spent on smartphone-based social media, daily time spent on smartphone-based information search, and daily time spent on smartphone-based entertainment)) (I) 0.14* (II) 0.16* (III) 0.15* (IV) 0.17* (V) − 0.19**	(I) S ( +) (II) S ( +) (III) S ( +) (IV) S ( +) (V) S (-)	Good (14)
30	Hou et al., 2021	China	723 (71.9%)	17 – 25, 19.96 (1.39)	Problematic Smartphone Use	MPAI (2.7 (0.71))	(I) Anxiety symptoms	SEM (Anxiety symptoms, Perceived social support) (I) 0.34***	(I) M ( +)	Fair (2, 14)
31	Jiang & Zhao, 2016	China	468 (55%)	18 – 24, 20.71 (1.47)	Problematic mobile phone use	PMPUS (Male: 39.43 ± 10.17; Female: 43.08 ± 9.66)	(I) Gender (being female) (II) Self-control Use patterns: (III) interpersonal (IV) transaction	SEM (Gender, self-control and mobile phone use patterns (Interpersonal, Entertainment, Transaction) (I) 0.13 ** (II) − 0.37 *** Use patterns: (III) 0.15 ** (IV) 0.14**	(I) S ( +) (II) M (-) Use patterns: (III) S ( +) (IV) S ( +)	Good (14)
32	Jiang & Zhao, 2017	China	468 (55%)	20.71 (1.47)	Problematic mobile phone use	PMPUS (Males: 39.43 ± 10.17, Females: 43.08 ± 9.66)	(I) Behavioral inhibition system (BIS) (II) Gender (being female)	HR (gender, time since acquisition, BAS, BIS and self-control) (I) -0.68*** (II) 0.11*	(I) L (-) (II) S ( +)	Fair (2, 14)
33	Jiang & Shi, 2016	China	630 (51%)	18 – 24, 20.63 (1.52)	Problematic mobile phone use	PMPUS (8.99%)	(I) Self-control (II) Self-esteem (III) Self-efficacy	LogR (Self-control, Self-esteem, Self-efficacy) (I) OR .899, 95% CI .869–.930 (II) OR 1.007, 95% CI .920–1.101 (III) OR 1.021, 95% CI .927–1.124	(I) VS (-) (II) VS ( +) (III) VS ( +)	Good (14)
34	Khoury et al., 2019	Brazil	415 (54.5%)	18 – 35, 23.6 (3.4)	Smartphone Addiction	SPAI (43.85%, ≥ 7)	(I) Facebook addiction (II) Anxiety disorders (III) Female gender (IV) Substance use disorders (V) Age between 18–25 years old (VI) Impulsivity (VII) Low satisfaction with social support	MR (Facebook addiction, Anxiety disorders, Female gender, Substance use disorders, Age, Impulsivity, Low satisfaction with social support) (I) OR 4.44, 95% CI 2.14–9.21 < 0.001 (II) OR 4.12, 95% CI 2.10–8.91 < 0.001 (III) OR 2.48, 95% CI 1.49–4.14 0.001 (IV) OR 2.48, 95% CI 1.29–4.77 0.007 (V) OR 1.09, 95% CI 1.01–1.19 0.021 (VI) OR 1.05, 95% CI 1.03–1.08 < 0.001 (VII) OR 1.03, 95% CI 1.01–1.99 0.016	(I) L ( +) (II) L ( +) (III) M ( +) (IV) M ( +) (V) VS ( +) (VI) VS ( +) (VII) VS ( +)	Good (14)
35	Kim et al., 2017	Korea	200 (63%)	19 – 28, 21.6 (2.0)	Smartphone addiction	SAPS	(I) Attachment avoidance (II) depression	SEM (Attachment anxiety, Attachment avoidance, Depression, Loneliness) (I) − 0.37* (II) 0.34**	(I) M (-) (II) M ( +)	Fair (2, 14)
36	Kim & Koh, 2018	Korea	313 (58.1%)	22 (3.4)	Smartphone addiction	SAPS (33.45 (7.67))	(I) Self-esteem (II) Anxiety	SEM (Anxiety, self-esteem, avoidant attachment) (I) -0.19* (II) 0.18*	(I) S (-) (II) S ( +)	Good (14)
37	Kim et al., 2019	Korea	608 (70%)	22.8	Smartphone addiction	SAPS (36% (40–43), 30.2% (≥ 44))	(I) stress (II) depression/anxiety symptom (III) suicidal ideation	LogR (Psychological health: stress, depression/anxiety symptom, suicidal ideation) (I) OR 2.19, 95% CI 1.55–3.10 *** (II) OR 1.91, 95% CI 1.27–2.86** (III) OR 2.24, 95% CI 1.52–3.31***	(I) M ( +) (II) S ( +) (III) M ( +)	Good (14)
38	Koç & Turan, 2021	Turkey	734 (61,4%)	19 – 25	Smartphone addiction	SDQ (2468 (0.777))	(I) SNS intensity (II) Self-esteem	SEM (SNS Intensity, self-esteem, subjective well being) (I) 0.643* (II) − 0.106*	(I) L ( +) (II) S (-)	Good (14)
39	Kuang-Tsan & Fu-Yuan, 2017	Taiwan	238 (55%)	18 – 22	Smart mobile phone addiction	MPAS (2.05)	(I) Gender (being female) (II) Academic stress (III) Love-affair stress	MR (Gender, Grade level, Academic stress, Stress of interpersonal relationship, Love-affair stress, Stress of self-career, Family life stress) (I) 0.119* (II) 0.145* (III) 0.371***	(I) S ( +) (II) S ( +) (III) M ( +)	Fair (2, 14)
40	Kuru & Celenk, 2021	Turkey	412 (63.6%)	18 – 35, 20.71 (2.52)	Smarphone addiction	SPAS-SV (29.50 (11.34))	Model 1: (I) Psychological inflexibility (II) Total effect anxiety (III) Direct effect anxiety Model 2: (I) Psychological inflexibility (II) Total effect depression	Mediation analyses (Model 1: Anxiety, psychological inflexibility; model 2: depression, psychological inflexibility) Model 1: (I) 0.183* (II) 0.168* (III) 0.133** Model 2: (I) 0.183* (II) 0.165*	Model 1: (I) S ( +) (II) S ( +) (III) S ( +) Model 2: (I) S ( +) (II) S ( +)	Fair (2, 14)
41	Laurence et al., 2020	Brazil	257 (72.8%)	22.4 (3.8)	Problematic smartphone use	Brazilian version of the smartphone addiction scale (SAS-BR) (98.00 (26.73))	(I) Loneliness Smartphone social app importance: (II) whatsapp importance (III) Instagram importance Smartphone model (IV) Others (Iphone, not samsung)	HMLR (Age, Sex, Family monthly income (BRL), smartphone social apps importance (Facebook, Whasapp, Instagram), loneliness, and smartphone model (samsung, others)) (I) 0.31*** Smartphone social app importance: (II) 0.26*** (III) 0.19** Smartphone model (IV) − 0.17**	(I) M ( +) (II) S ( +) (III) S ( +) Smartphone model (IV) S (-)	Good
42	Lian & You, 2017	China	682 (44.6%)	18 – 24, 19.34 (1.26)	Smarphone addiction	MPAI (2.89 (0.66), 52,9% (scored highest 27%))	(I) Conscientiousness (II) Relationship virtues (III) Vitality virtues	MR (Age, gender, conscientiousness, relationship virtue, vitality virtue) (I) − 0.20*** (II) 0.10* (III) 0.11*	(I) S (-) (II) S ( +) (III) S ( +)	Fair (2, 14)
43	Lian, 2018	China	706 (46.8%)	18 – 24, 19.62 (1.21)	Smarphone addiction	MPAI (2.64 (0.67))	(I) Conscientiousness (II) Interpersonal	MR (Virtues (Interpersonal, Vitality, Conscientiousness)) (I) − 0.22*** SEM (two virtues (Conscientiousness, interpersonal), alienation) (II) -0.23**	(I) S (-) (II) S (-)	Fair (2, 14)
44	Lin et al., 2021	China	863 (59%)	17 – 23, 20.93	Smarphone addiction	SAS-C (2.85 (0.55)	(I) Time (II) Grade (III) Interpersonal sensitivity (IV) FoMO	Moderated mediation effect analysis (Control variables: gender, time, grade, major, if the one-child, parenting style, growth environment; Independent variable: interpersonal sensitivity; Mediator: FoMO) (I) 0.30* (II) 0.05* (III) 0.35** (IV) 0.24**	(I) M ( +) (II) VS ( +) (III) M ( +) (IV) S ( +)	Good (2)
45	Lin & Chiang, 2017	Singapore	438 (53%)	22.29 (1.63)	Smartphone dependency	MPAI (NR)	(I) Gender (being female) Psychological attributes: (II) leisure boredom Smartphone activities: (III) mobile social media, (IV) mobile gaming, (V) mobile videos, (VI) traditional phone use	SEM (Leisure boredom, Sensation seeking, The use of mobile social media, The use of mobile gaming, The use of mobile videos, The use of traditional phone activities) (I) -0.203*** Psychological attributes: (II) 0.238*** Smartphone activities: (III) 0.091*, (IV) 0.142**, (V) 0.170***, (VI) 0.091*	(I) S (-) (II) S ( +) (III) VS ( +) (IV) S ( +) (V) S ( +) (VI) VS ( +)	Fair (3, 14)
46	Liu et al., 2020	China	1169 (43.8%)	17 – 23, 19.89 (1.25)	Smarphone addiction	MPAI (2.69 (0.70))	(I) Neuroticism (II) Childhood psychological maltreatment (III) Negative coping style	SEM (neuroticism, negative coping style, childhood psychological maltreatment) (I) 0.19*** (II) 0.16*** (III) 0.40***	(I) S ( +) (II) S ( +) (III) M ( +)	Good (14)
47	Liu et al., 2021	China	908 (52%)	17 – 27, 21.04 (1.84)	Mobile Phone Dependence	PMPUQ and MPAI (2.72 (0.83))	Mediation analysis: (I) Attachment anxiety (II) Loneliness Moderated mediation analysis: (I) Attachment anxiety (II) Loneliness (III) Rumination	Mediation analysis (Gender, Age, Attachment anxiety, Loneliness) (I) 0.16*** (II) 0.33*** Moderated mediation analysis (Gender, Age, Attachment anxiety, Loneliness, Rumination, Attachment Anxiety × Rumination, Loneliness × Rumination) (I) 0.16*** (II) 0.20*** (III) 0.18***	Mediation analysis: (I) S ( +) (II) M ( +) Moderated mediation analysis (I) S ( +) (II) S ( +) (III) S ( +)	Good (14)
48	Long et al., 2016	China	1062 (54%)	17 – 26, 20.65 (1.54)	Problematic smartphone use	PCPUQ (21.3%, ≥ 4 of the first 7 questions and any of the last 5 questions)	(I) science-humanities division (majoring in the humanities) (II) monthly income from the family (high monthly income from the family (≥ 1500 RMB)) (III) emotional symptoms (IV) perceived stress (V) perfectionism-related factors (high doubts about actions) (VI) perfectionism-related factors (high parental expectations)	LogR (Gender, grade, science-humanities division, monthly income from the family, and all psychological risk factors (SAS Zung Self-Rating Anxiety Scale, SDS Zung Self-Rating Depression Scale, PSS Perceived Stress Scale, CFMPS-DA Chinese Frost Multidimensional Perfectionism Scale-Doubts about Actions subscale, CFMPS-PE CFMPS-Parental Expectations subscale, CFMPS-CM CFMPS- Concern over Mistakes subscale, CFMPS-OR CFMPS-Organization subscale, CFMPS-PS CFMPS- Personal Standards subscale)) (I) AOR 2.14, 95% CI 1.45–3.16 (II) AOR 2.45, 95% CI 1.46–4.13 (III) AOR 1.01, 95% CI 1.01–1.03 (IV) AOR 1.06, 95% CI 1.02–1.10 (V) AOR 1.15, 95% CI 1.08–1.22 (VI) AOR 1.04, 95% CI 1.00–1.08	(I) M ( +) (II) M ( +) (III) VS ( +) (IV) VS ( +) (V) VS ( +) (VI) VS ( +)	Good (14)
49	Matar Boumosleh & Jaalouk, 2017	Líbano	688 (47%)	20.64 (1.88)	Smartphone addiction	SPAI (55.37 (15.04))	Depression: (I) depression score (II) personality type A (III) excessive smartphone use (≥ 5 h/ weekday) (IV) non-use of smartphone for calling family members (VI) use of smartphone for entertainment purposes Anxiety: (I) anxiety score (II) personality type A (III) excessive smartphone use (IV) non-use of smartphone to call family members (V) use of smartphone for entertainment purposes	MLR (depression/anxiety, age, personality type, class, age at first use of smartphone, duration of smartphone use, and use of smartphone for calling family members, entertainment and other purposes) Depression: (I) 0.201*** (II) 0.130* (III) 0.262*** (IV) -0.148* (VI) 0.126* Anxiety: (I) 0.122* (II) 0.132* (III) 0.268* (IV) -0.160** (V) 0.125*	Depression: (I) S ( +) (II) S ( +) (III) S ( +) (IV) S (-) (VI) S ( +) Anxiety: (I) S ( +) (II) S ( +) (III) S ( +) (IV) S (-) (V) S ( +)	Good (14)
50	Pourrazavi et al., 2014	Iran	476 (60%)	18 – 33	Mobile phone problematic use	MPPUS (25.5%, NR)	(I) Self-efficacy to avoid EMPU (II) Observational learning (III) Self-regulation (IV) Self-control (V) Attitude toward EMPU (VI) EMPU (excessive mobile phone use)	MLR (Excessive mobile phone use, social cognitive theory constructs (self-efficacy, outcome expectation, and self-regulation), attitude, and self-control) (I) -0.34*** (II) 0.15*** (III) -0.09* (IV) -0.15*** (V) -0.20*** (VI) 0.09*	(I) M ( +) (II) S ( +) (III) VS ( +) (IV) S ( +) (V) S ( +) (VI) VS ( +)	Good (14)
51	Roberts & Pirog III, 2013	USA	191 (41%)	19 – 38, 21 (1)	Mobile phone addiction	MPAT (5.093 (1.272))	(I) Materialism (II) Impulsiveness	PM (Impulsiveness, Materialism) (I) .332*** (II) .152*	(I) M ( +) (II) S ( +)	Fair (2, 14)
52	Roberts et al., 2015	USA	346 (51%)	19 – 24, 21	Cell phone addiction	MRCPAS (NR)	(I) Emotional instability (II) Introversion (III) Materialism (IV) Attention impulsiveness	Hierarchical model (Seven personality factors: emotional instability, introversion, openness to experience, agreeableness, conscientiousness, materialism, and need for arousal) (I) 0.20** (II) 0.15* (III) 0.13** (IV) 0.22*	(I) S ( +) (II) S ( +) (III) S ( +) (IV) S ( +)	Fair (2, 14)
53	Rozgonjuk et al., 2019	USA	261 (77%)	19.73 (3.52)	Problematic smartphone use	SAS-SV (26.31 (10.35))	(I) Social smartphone use (II) Non social smartphone use	SEM (Social smartphone use, Non social smartphone use, Intolerance of uncertainty) (I) 0.159* (II) 0.392***	(I) S ( +) (II) M ( +)	Fair (2, 14)
54	Rozgonjuk & Elhai, 2021	USA	300 (76%)	18 – 38, 19.45 (2.17)	Problematic smartphone use	SAS (93.47 (25.30))	(I) Age (II) Gender (being female) (III) Process smartphone use	SEM (Age, Gender, Expressive suppression, social smartphone use, process smartphone use) (I) -0.114* (II) 0.153** (III) 0.556***	(I) S (-) (II) S ( +) (III) L ( +)	Good (14)
55	Salehan & Negahban, 2013	USA	214 (39%)	90% 18—30	Mobile addiction	SDQ (NR)	(I) Use of mobile social networking applications	SEM (Use of mobile social networking applications, SNS intensity, gender, network size) (I) 0.51*	(I) L ( +)	Fair (2, 14)
56	Sun et al., 2021	China	800 (60%)	19.06 (1.35)	Problematic smartphone use	MPATS (2.634 (0.699))	Mediation Model: (I) Age (II) Ostracism (III) Social self-efficacy Moderated Mediation Model: (IV) Rejection sensitivity (RS)	Mediation Model (Gender, Age, Ostracism, Social self-efficacy) (I) 0.063* (II) 0.219*** (III) − 0.124** Moderated Mediation Model (Gender, Age, Ostracism, Social self-efficacy, Rejection sensitivity (RS), Ostracism × RS) (IV) 0.313***	(I) VS ( +) (II) S ( +) (III) S (-) (IV) M ( +)	Good (14)
57	Takao, 2014	Japan	396 (78%)	18 – 25, 20.07 (1.35)	Problematic mobile phone use	MPPUS (103.7 (38.88))	(I) Gender (being female) (II) Extraversion (III) Low neuroticism (IV) Openness	MR (Gender and five personality domains (extreversion, neuroticism, openness, agreeableness and conscientiousness) (I) 0.12** (II) 0.24*** (III) -0.23*** (IV) 0.18***	(I) S ( +) (II) S ( +) (III) S (-) (IV) S ( +)	Fair (2, 14)
58	Wolniewicz et al., 2020	USA	297 (72%)	19.70 (3.96)	Problematic smartphone use	SAS (91.52 (23.95))	(I) Age (II) FoMO (III) SUF	SEM (age and sex (control variables), depression and anxiety severity (predictor variables), FOMO and boredom proneness as mediators (with boredom proneness statistically predicting FOMO)) (I) -0.26*** (II) 0.58*** (III) 0.19***	(I) S (-) (II) L ( +) (III) S ( +)	Good (14)
59	Xiao et al., 2021	China	1267 (59%)	18 – 30, 20.36 (0.97)	Problematic mobile phone use	MPAI (2.78 (0.72))	(I) alexithymia (II) social interaction anxiousness (III) boredom proneness	Model 6 of SPSS PROCESS macro (multiple mediation model) (alexithymia, boredom proneness, social interaction anxiousness) (I) 0.25*** (II) 0.29*** (III) 0.19***	(I) S ( +) (II) S ( +) (III) S ( +)	Fair (2, 14)
60	Yang et al., 2019	China	608 (74%)	20.06 (1.98)	Mobile phone dependence	MPATS (42.81 (10.63))	(I) physical exercise (II) self-control	Mediating Role Analysis (physical exercise, self-control) (I) -0.131** (II) -0.557***	(I) S (-) (II) L (-)	Good (14)
61	Yang et al., 2021a, 2021b	China	608 (74%)	20.06 (1.98)	Mobile phone addiction	MPATS (78.29 (32–56), 8.06 (≥ 57))	(I) Gender (II) major (III) physical activity (PA) (IV) W1, separated net programs (V) W2, confrontation programs	PROCESS macro (Model 1) (Gender, major, physical activity, W1, separated net programs; W2, confrontation programs; W3, difficulty beauty programs; PA*W1; PA*W2; PA*W3) (I) 0.271* (II) − 0.169* (III) − 0.266*** (IV) 0.263* (V) 0.445*	(I) S ( +) (II) S (-) (III) S (-) (IV) S ( +) (V) M ( +)	Good (14)
62	Yang et al., 2020a, 2020b	China	1099 (59.6%)	20.04 (1.25)	Problematic mobile phone use	MPAI (2.63 (0.63))	(I) Gender (being female) (II) Age (III) Boredom proneness (IV) Depression	Mediation analysis (Gender, Age, Boredom proneness, Depression) (I) 0.44*** (II) − 0.05 (III) 0.27*** (IV) 0.17***	(I) M ( +) (II) VS (-) (III) S ( +) (IV) S ( +)	Fair (2, 14)
63	You et al., 2019	China	653 (54%)	17 – 25, 19.94 (1.34)	Mobile phone addiction	Mobile phone addiction scale (Xiong et al., 2012) (2.73 ± 0.69)	(I) Gender (II) socio-economic status (III) interpersonal sensitivity	SEM (Gender, age, socio-economic status, social anxiety, self-esteem, interpersonal sensitivity) (I) 0.10* (II) 0.10** (III) 0.29**	(I) S ( +) (II) S ( +) (III) S ( +)	Good (2)
64	Yuchang et al., 2017	China	297 (45%)	17 – 24, 20.24 (1.08)	Smartphone Addiction	SAS-SV (23.75 (7.47), 27.92% (≥ 31 M, ≥ 33 F))	(I) Anxiety (II) self-esteem	SEM (Anxiety attachment dimension, depend attachment dimension, close attachment dimension, dysfunctional attitudes, self-esteem) (I) -0.218** (II) − 0.357**	(I) S (-) (II) M (-)	Fair (2, 14)
65	Zhang et al., 2020b	China	764 (59%)	19.83 (1.10)	Problematic Smartphone Use	MPAI (2.66 (0.58))	(I) Interpersonal adaptation	Moderated mediation analysis (Control variables: gender, grade; Predictor: parental attachment; Mediator: interpersonal adaptation; Moderator: self − control; Interaction: interpersonal adaptation × self-control) (I) − 0.10**	(I) S (-)	Good (2)
66	Zhang et al., 2020a	China	1304 (60%)	18 – 22, 19.71 (1.03)	Smartphone use disorder	MPAI (1.99 (0.58))	(I) Future time perspective (II) Depression	MLR (future time perspective (FTP), depression) (I) -0.13*** (II) 0.70***	(I) S (-) (II) L ( +)	Good (14)
67	Zhu et al., 2019	China	356 (64%)	17 – 19, 18.33 (0.57)	Smartphone use disorder	MPAI (2.51 (0.64))	(I) perceived discrimination (II) school engagement (III) parental rejection	Mediation Model Test (perceived discrimination, school engagement, parental rejection) (I) -0.19** (II) -0.16** (III) 0.14*	(I) S (-) (II) S (-) (III) S ( +)	Fair (2, 14)
Problematic social media use (PSMU)										
Prospective cohort studies										
68	Brailovskaia et al., 2018	Germany	122 (82.8%)	17 – 38, 21.70 (3.67)	Facebook Addiction Disorder	BFAS (8.98 (3.64))	(I) Physical activity	Bootstrapped mediation analysis (Physical activity, daily stress) (I) -0.796*	(I) L (-)	Good (14)
69	Brailovskaia & Margraf, 2017	Alemania	179 (77%)	17 – 58, 22.52 (5.00)	Facebook addiction disorder	BFAS (9.77 (3.86))	(I) Narcissism	Bootstrapped mediation analysis (Narcissism, Stress symptoms) (I) 0.259*	(I) S ( +)	Good (14)
Cross-sectional studies										
70	Aladwani & Almarzouq, 2016	Kuwait	407 (46%)	20.04 (1.16)	Compulsive social media use	CIUS (2.51 (0.71))	(I) Self-esteem (II) Interaction anxiousness	PM (Self-esteem, Interaction anxiousness) (I) − 0.22* (II) 0.24**	(I) S (-) (II) S ( +)	Fair (2, 14)
71	Balcerowska et al., 2019	Poland	486 (64%)	21.56 (4.50)	Facebook addiction	BFAS (12.88 (4.93))	(I) Gender (II) Admiration demand (III) Self-sufficiency	MHR (Gender, age, Big Five personality traits (Neuroticism, Extraversion, Openness to experience, Agreeableness, Conscientiousness), and four dimensions of narcissism (Lead-ership, Vanity, Self-sufficiency and Admiration Demand)) (I) –0.23*** (II) 0.37*** (III) –0.18***	(I) S (-) (II) M ( +) (III) S (-)	Good (14)
72	Casale et al., 2018	Italy	579 (54.6%)	22.39 (2.82)	Social media problematic use	BSMAS (11.96 (4.99))	Females: (I) fear of missing out (II) self-presentational social skills (III) positive metacognitions Males: (I) fear of missing out (II) positive metacognitions	SEM (Fear of negative evaluation, Fear of missing out, Self-presentational skills, Positive metacognitions) Females: (I) 0.34*** (II) 0.38*** (III) 0.42* Males: (I) 0.38* (II) 0.28*	Females: (I) M ( +) (II) M ( +) (III) M ( +) Males: (I) M ( +) (II) S ( +)	Fair (2, 14)
73	Casale & Fioravanti, 2018	Italy	535 (50.08%)	22.70 (2.76)	Facebook addiction	BFAS (1.67 (0.64))	(I) Need to belong (II) Need for admiration	SEM (effects of grandiose and vulnerable narcissism on Fb addiction levels via the need to be admired and the need to belong) (I) 0.38* (II) 0.26*	(I) M ( +) (II) S ( +)	Fair (2, 14)
74	Casale & Fioravanti, 2017	Italy	590 (53.2% female)	22.29 (2.079)	Problematic Social Networking Sites Use	GPIUS-2 (33.14 (16.03))	(I) Experiences of Shame (II) Escapism (III) Control over self-presentation (IV) Approval/acceptance	SEM (effects of shame on problematic SNS use through perceived relevance/benefits of CMC (i.e. control over self-presentation, escapism, and approval/acceptance) (I) 0.44* (II) 0.15* (III) 0.11* (IV) 0.27*	(I) M ( +) (II) S ( +) (III) S ( +) (IV) S ( +)	Fair (2, 14)
75	Chung et al., 2019	Malaysia	128 (52%)	18 – 29, 19.73 (1.99)	Social media addiction	BSMAS (16.74 (4.16))	(I) Gender (being a female) (II) Social media usage (III) Psychopathy	HMR ( Age, gender, social media usage, impulsivity, and the Dark Tetrad traits (Machiavellianism, narcissism, psychopathy, and sadism) (I) − 0.18* (II) 0.19* (III) 0.28**	(I) S (-) (II) S ( +) (III) S ( +)	Good (14)
76	Demircioğlu & Köse, 2020	Turkey	400 (66%)	18 – 42, 21.36 (2.20)	Social Media Addiction	Social Media Addiction Scale (2.18 (0.70))	(I) Fearful attachment (II) Preoccupied attachment (III) Self-esteem	SEM (Fearful attachment, Preoccupied attachment, Secure attachment, Self-esteem; moderator: Gender) (I) 0.012* (II) 0.12** (III) -0.27***	(I) VS ( +) (II) S ( +) (III) S (-)	Fair (2, 14)
77	Demircioğlu & Göncü Köse, 2021	Turkey	229 (68%)	18 – 32, 21.51 (1.80)	Social Media Addiction	Social Media Addiction Scale (2.15 (0.70))	(I) Relationship satisfaction (II) fearful attachment (III) rejection sensitivity (IV) psychopathy	SEM (Relationship satisfaction, Attachment styles (Secure Attach, Fearful Attach, Preoccupied Attach, Dismissive Attach), Rejection Sensitivity, Dark triad personality traits (Narcissism, machiavellianism, psychopathy)) (I)—0.16* (II) 0.14* (III) 0.15* (IV) 0.17*	(I) S (-) (II) S ( +) (III) S ( +) (IV) S ( +)	Fair (2, 14)
78	Dempsey et al., 2019	USA	291 (57.6%)	18—25 (20.03 (3.06))	Problematic Facebook Use	BFAS (11.33 (5.06))	(I) Age (II) FoMO (III) Rumination (IV) Facebook use frequency	SEM (age and gender as covariates; FoMO, Rumination, depression severity, social anxiety, life satisfaction, Facebook use frequency) (I) 0.14* (II) 0.26*** (III) 0.13* (IV) − 0.35***	(I) S ( +) (II) S ( +) (III) S ( +) (IV) M (-)	Fair (2, 14)
79	Duran, 2015	Spain	199 (72%)	18–22 (females: 19.09 (1.29), males: 19.19 (1.28))	Tuenti addiction	CERI (1.44 (0.79))	(I) Comunicación Privada (II) Actitud positiva hacia la aceptación madre como contacto Tuenti	Análisis de regresión jerárquica (usos de la red social Tuenti, actitudes hacia la aceptación de los padres, y género) (Paso 1: Comunicación Privada, Aceptación madre; Paso 2: Comunicación Privada x Género) (I) 0.20* (II) -0.16*	(I) S ( +) (II) S (-)	Good (14)
80	Foroughi et al., 2021	Malaysia	364 (51.1%)	19 – 26	Instagram addiction	BFAS (NR)	(I) recognition needs (II) social needs (III) entertainment needs	SEM (Academic performance, depression, entertainment needs, information needs, Instagram addiction, life satisfaction, recognition needs, social anxiety, social needs, physical activity) (I) 0. 295** (II) 0.243** (III) 0.207**	(I) S ( +) (II) S ( +) (III) S ( +)	Fair (2, 14)
81	Gao et al., 2021	China	849 (47%)	19.0 (1.36)	Excessive WeChat use	Excessive WeChat Use scale (2.54 ( 0.76))	(I) Depression (II) Anxiety (III) WeChat use intensity	Mediation analysis (Psychological needs satisfaction, anxiety, depression and WeChat use intensity) (I) 0.184*** (II) 0.194*** (III) 0.515***	(I) S (-) (II) S (-) (III) L ( +)	Fair (2, 14)
82	Hong et al., 2014	China	215 (46%)	18 – 22	Facebook addiction	IAT (Withdrawal 5.55 (2.90); Tolerance 9.26 (3.58); Life problems 7.29 (3.29); Substitute satisfaction 8.49 (3.33))	(I) Depressive character (II) Facebook usage	SEM (self-esteem, social extraversion, sense of self-inferiority, neuroticism, and depressive character, the mediating variable was Facebook usage) (I) 0.21* (II) 0.62***	(I) S ( +) (II) L ( +)	Fair (2, 14)
83	Hong & Chiu, 2016	China	206 (53%)	18 – 22	Facebook addiction	IAT (Withdrawal and tolerance 6.37 (3.30); Life problems 5.93 (2.91); Substitute satisfaction 8.86 (3.66))	(I) Online psychological privacy (II) Facebook usage motivation (III) Facebook usage	SEM (online psychological privacy, Facebook usage motivation, Facebook usage) (I) 0.302*** (II) 0.439*** (III) 0.296***	(I) M ( +) (II) M ( +) (III) S ( +)	Fair (2, 14)
84	Hou et al., 2017a	China	1245 (52%)	Sample 1: 20.7 (2.1); Sample 2: 19.8 (1.3)	WeChat Excessive Use	WeChat Excessive Use Scale (WEUS) (13.6% (15.1–21.4), 8.2% (21.4–27.7), 6.6% (> 27.7))	(I) External locus of control (II) Online social interaction	Mediation analysis (external locus of control, online social interaction) (I) 0.14* (II) 0.30*	(I) S ( +) (II) M ( +)	Good (14)
85	Hou et al., 2017b	China	499 (77.6%)	19.90 (1.35)	Problematic SNS usage	FIQ (19.61 (6.15))	(I) Gender (being female) (II) Perceived stress (III) Psychological resilience	HMR (Age, gender, Perceived stress, Psychological resilience, Stress ∗ resilience) (I) 0.16** (II) 0.21** (III) -0.09***	(I) S ( +) (II) S ( +) (III) VS (-)	Good (14)
86	Hou et al., 2019	China	641 (74.4%)	19.90 (1.37)	Problematic SNS usage	FIQ (19.43 (7.14))	(I) Age (II) Depression (III) Anxiety	Moderated mediation model (Gender, age, Perceived stress, Depression, Anxiety) (I) 0.14** (II) 0.14* (III) 0.12*	(I) S ( +) (II) S ( +) (III) S ( +)	Good (14)
87	Jaradat & Atyeh, 2017	Jordan	380 (72.9%)	86% 20 – 25	Social Media Addiction	IAT (Withdrawal 2.63 (1.05); Tolerance 3.06 (1.20); Life problems 3.12 (0.96)); Substitute satisfaction 2.98 (0.10))	(I) Neuroticism (II) Openness (III) Extraversion	Hypothetical model (the relationships among the five personality trait factors (neuroticism, openness, extraversion, agreeableness, conscientiousness), Social media addiction and the moderator variables Gender, Age, College Expense and Experience) (I) -0.244*** (II) 0.182*** (III) 0.150***	(I) S (-) (II) S ( +) (III) S ( +)	Good
88	Jasso-Medrano & Lopez-Rosales, 2018	Mexico	374 (58.6%)	18 – 24, 20.01 (1.84)	Addiction to social media	Social Network Addiction Questionnaire (2.33 (0.71))	(I) Frequency of the use of mobile devices (II) Daily hours of use (III) Suicidal ideation (IV) Depression	SEM (Daily hours, mobile use, suicidal ideation, depression) (I) 0.21*** (II) 0.42*** (III) -0.21** (IV) 0.46***	(I) S ( +) (II) M ( +) (III) S (-) (IV) M ( +)	Fair (2, 14)
89	Kircaburun & Griffiths, 2018	Turkey	752 (69%)	18 – 24, 20.30 (1.46)	Instagram addiction	IAT (26.5% (38–58), 6.1% (59–73), 0.9% (> 73))	(I) Agreeableness (II) Self-liking (III) Daily Internet use	SEM (self-liking between Instagram addiction and the Big Five personality dimensions (neuroticism, openness, extraversion, agreeableness, conscientiousness)) (I) − 0.17** (II) − 0.14** (III) 0.20**	(I) S (-) (II) S (-) (III) S ( +)	Fair (2, 14)
90	Kircaburun et al., 2020a, 2020b	Turkey	460 (61%)	18 – 26, 19.74 (1.49)	Problematic social media use	Social Media Use Questionnaire (24.10 (6.73))	(I) self-confidence (II) self/everyday creativity (III) depression	SEM (task-oriented, self-confidence, risk-taking, self/everyday creativity, depression, loneliness, internal motivation, loneliness) (I) − 0.16* (II) − 0.23* (III) 0.23**	(I) S (-) (II) S (-) (III) S ( +)	Fair (2, 14)
91	Kircaburun et al., 2020b	Turkey	1008 (60.5%)	17 – 32, 20.49 (1.73)	Problematic social media use	Social Media Use Questionnaire (15.21 (7.48))	(I) Gender (being female) (II) neuroticism (III) agreeableness (IV) extraversion (V) conscientiousness (VI) Instagram use (VII) Snapchat use (VIII) Facebook use (IX) Passing time (X) Maintaining existing relationships (XI) Meeting new people and socializing (XII) entertainmental use (XIII) informational and educational use	HR (gender, age, personality traits (neuroticism, openness, extraversion, agreeableness, conscientiousness), most used social media platforms (Facebook, Instagram, Whatsapp, Twitter, Scapchat, Youtube, Google), and social media use motives (maintaining existing relationships, meet new people and socializing, make, express, or present more popular oneself, pass time, as a task management tool, entertainmental, informational and educational)) (I) − 0.19*** (II) 0.10*** (III) 0.06* (IV) − 0.08** (V) − 0.06* (VI) 0.10*** (VII) 0.08* (VIII) 0.06* (IX) 0.27*** (X) 0.12*** (XI) 0.12*** (XII) 0.08* (XIII) − 0.07*	(I) S (-) (II) S ( +) (III) VS ( +) (IV) VS (-) (V) VS (-) (VI) S ( +) (VII) VS ( +) (VIII) 0.06* (IX) S ( +) (X) S ( +) (XI) S ( +) (XII) VS ( +) (XIII) VS (-)	Fair (2, 14)
92	Lee, 2019	Malaysia	204 (60%)	18 – 27, 22.94 (3.43)	SNS addiction	BFAS (NR)	(I) Age (II) Gender (being female) (III) Openness (IV) Psychopaty	HR (Age, gender, five-factor model (extraversion, agreeableness, conscientiousness, neuroticism, openness), dark triad (psychopaty, machiavellianism,narcissism)) (I) -0.14* (II) -0.17* (III) -0.20** (IV) 0.23*	(I) S (-) (II) S (-) (III) S (-) (IV) S ( +)	Fair (2, 14)
93	Marino et al., 2016	Italy	815 (77%)	18 – 35, 21.17 (2.15)	Problematic Facebook use	GPIUS-2 (28.74 (14.12))	(I) coping (II) conformity (III) enhancement (IV) extraversion (V) negative beliefs about thoughts (VI) cognitive confidence	PM (Personality traits (agreeableness, conscientiousness, emotional stability, extraversion, and openness), Motives for using Facebook (coping, conformity, enhancement, and social motive) and metacognitions (positive beliefs about worry, negative beliefs about thoughts, lack of cognitive confidence, beliefs about the need to control thoughts and cognitive self-consciousness)) (I) 0.42** (II) 0.28** (III) 0.17** (IV) 0.09* (V) 0.12** (VI) 0.08*	(I) M ( +) (II) S ( +) (III) S ( +) (IV) VS ( +) (V) S ( +) (VI) VS ( +)	Good (14)
94	Punyanunt-Carter et al., 2018	USA	396 (71%)	21.51 (2.41)	Social media addiction	BFAS (NR)	(I) Introversion (II) Social media Communication Apprehension	ML (Introversion, Social media Communication Apprehension) (I) -0.12* (II) 0.17**	(I) S (-) (II) S ( +)	Fair (2, 14)
95	Raza et al., 2020	Pakistan	280 (60%)	90% 18 – 27	Intensive Facebook usage	Items adapted from Su and Chan (2017) (NR)	(I) Information seeking (II) Subjective norms (III) Social relationship	PLS-SEM (Uses and gatifications theory: escape, information seeking, ease of use, social relationship, career opportunities, and education; theory of planned behavior: social influence, perceived behavioral, control, and attitude) (I) 0.105* (II) 0.229*** (III) 0.167***	(I) S ( +) (II) S ( +) (III) S ( +)	Fair (2, 14)
96	Satici & Uysal, 2015	Turkey	311 (58%)	18 – 32, 20.86 (1.61)	Problematic Facebook use	BFAS (32.43 (14.83))	(I) life satisfaction (II) subjective vitality (III) flourishing	MR (life satisfaction, flourishing, subjective happiness, and subjective vitality) (I) -0.18** (II) -0.15* (III) -0.15**	(I) S (-) (II) S (-) (III) S (-)	Good (14)
97	Sayeed et al., 2020	Bangladesh	405 (49%)	21.03 (1.94)	Facebook addiction	BFAS (36.9%)	(I) domestic violence (II) sleeping more than 6–7 h per day (III) depressive symptoms (IV) spending 5 h or more per day using Facebook	BLogR (University, Failure in love, Domestic violence, Smoking history, Sleeping status, Drug addiction, Depression status, Stressful life event, Facebook use per day, Facebook use for educational purposes, Online shopping) (I) AOR 2.519; 95% CI: 1.271–4.991; *p* < 0.01 (II) AOR 2.112; 95% CI: 1.246–3.582; p < 0.01 (III) AOR 1.667; 95% CI: 1.055–2.634; *p* < 0.05 (IV) AOR 1.670; 95% CI: 1.063–2.623; p < 0.05	(I) M ( +) (II) M ( +) (III) S ( +) (IV) S ( +)	Good (14)
98	Shan et al., 2021	China	607 (63%)	18 – 23, 19.24 (1.01)	Social Networking Sites Addiction	Social Networking Sites Addiction Scale (2.85 (0.70))	Model 1 (I) Rejection sensitivity (II) Psychological capital Model 2 (I) Rejection sensitivity (II) Fearful avoidant style Model 5 (I) Rejection sensitivity (II) Psychological capital (III) Secure style	Model 1. Multiple mediation regression analysis (Rejection sensitivity, Psychological capital, Attachment styles) (I) 0.152*** (II) − 0.151*** Model 2. Multiple mediation regression analysis (Rejection sensitivity, Psychological capital, Fearful avoidant style) (I) 0.134*** (II) 0.322*** Model 5. Multiple mediation regression analysis (Rejection sensitivity, Psychological capital, Secure style) (I) 0.152*** (II) -0.151*** (III) -0.166***	Model 1 (I) S ( +) (II) S (-) Model 2 (I) S ( +) (II) M ( +) Model 5. (I) S ( +) (II) S (-) (III) S (-)	Good (14)
99	Sheldon et al., 2021	USA	337 (57%)	23.35 (8.08)	Facebook addiction, Instagram addiction, Snapchat addiction	BFAS, replacing the word “Facebook” (1.91 (0.73)) with “Instagram” (2.26 (0.93)) and “Snapchat” (2.08 (0.94)) for those platforms, respectively	Facebook addiction: (I) FOMO Instagram addiction: (I) FOMO Snapchat addiction: (I) FOMO (II) Social activity	Facebook addiction: HLR (FOMO, Interpersonal interaction, Life satisfaction) (I) 0.35*** Instagram addiction: HLR (FOMO, conscientiousness, extraversion) (I) 0.43*** Snapchat addiction: HLR (FOMO, extraversion, social activity) (I) 0.40*** (II) 0.13*	Facebook addiction: (I) M ( +) Instagram addiction: (I) M ( +) Snapchat addiction: (I) M ( +) (II) S ( +)	Good (14)
100	Siah et al., 2021	Malaysia	219 (57%)	19 – 25, 21.46 (1.17)	Social Media Addiction	BSNAS (NR)	(I) Narcissism (II) Avoidance (III) Gender	SEM (Dark Triad Personalities (Machiavellianism,narcissism, psychopathy), Coping Strategies (avoidance, positive thinking, roblem solving, social support)) (I) 0.17* (II) 0.25* (III) 0.15***	(I) S ( +) (II) S ( +) (III) S ( +)	Fair (2, 14)
101	Süral et al., 2019	Turkey	444 (75%)	18 – 43, 20.45 (3.57)	Problematic social media use	SMUQ (2.71 (0.75))	(I) Trait emotional intelligence (TEI) (II) “maintain my existing relationships” (III) “meet new people and socialize” (IV) “express or present myself as being more popular” (MEPO) (V) “pass time” (PT) (VI) “entertain myself” (V) “manage my tasks and media (videos, photos, etc.)”	PM (Trait emotional intelligence, Social Media Use Motives (“maintain my existing relationships”, “meet new people and socialize”, “express or present myself as being more popular”, “pass time”, (v) “entertain myself”, “manage my tasks and media (videos, photos, etc.)”, and “access information and education”)) (I) − 0.39*** (II) 0.09* (III) 0.13** (IV) 0.13** (V) 0.13* (VI) 0.11* (V) 0.08*	(I) M (-) (II) VS ( +) (III) S ( +) (IV) S ( +) (V) S ( +) (VI) S ( +) (V) VS ( +)	Fair (2, 14)
102	Uysal, 2015	Turkey	229 (52%)	18 – 27, 21 (1.64)	Problematic Facebook use	BFAS (30.09 (10.21))	(I) Social safeness (II) Flourishing	MHR (Age, gender, social safeness, flourishing, internet usage time) (I) -0.29** (II) -0.24**	(I) S (-) (II) S (-)	Good (14)
103	Varchetta et al., 2020	Italy	306 (50%)	18 – 30, 21.80 (3.19)	Social Media Addiction	BSMAS (2.21 (0.81)	(I) FOMO (II) frequency of social network use during the main daily activities (Social Media Engagement Scale, SMES)	LR (FOMO, SMES) (I) 0.61*** (II) 0.27***	(I) L ( +) (II) S ( +)	Fair (2, 14)
104	Xie & Karan, 2019	USA	526 (41%)	18 – 29, 24.21 (5.92)	Facebook addiction	BFAS (3.15 (0.71))	(I) Facebook use intensity (II) Facebook use for broadcasting (III) Trait anxiety	HR (Block 1: age, gender, education, household income, white; Block 2: trait anxiety; Block 3: Facebook use intensity; Block 4: Facebook activities (Broadcasting, directed communication); Block 5: Gender × Trait anxiety) (I) 0.57*** (II) 0.20** (III) 0.12*	(I) L ( +) (II) S ( +) (III) S ( +)	Good (3)
105	Yu & Luo, 2021	China	390 (55%)	19.09 (1.47)	Social Networking Addiction	SMD (2.80 (2.21))	(I) Reactive restriction (II) Limiting online behaviors	LogR (Block 1: Gender, age; Block 2: Reactive restriction, internet-specific rules, limiting online bhaviors, quality of communication) (I) OR 1.78; 95%CI = 1.24–2.55 (II) OR 1.72; 95%CI = 1.11–2.65	(I) S ( +) (II) S ( +)	Good (14)
106	Yu & Chen, 2020	Taiwan	316 (72%)	20.95 (2.70)	Social Networking Addiction	BSMAS (NR)	(I) frequency of Facebook Stories updates (II) time spent reading Facebook Stories	MIMIC (Frequency of Facebook Stories updates, frequency of news feed updates, time spent reading Facebook Stories, time spent reading Facebook news feeds) (I) 0.49* (II) 0.13*	(I) L ( +) (II) S (-)	Fair (2, 14)
Internet Gaming Disorder (IGD)										
Prospective cohort studies										
107	Dang et al., 2019	China	283 (60%)	18 – 27, 20.47 (1.15)	IGD	DSM-5 IGD scale (1.45 (1.97))	(I) depression (W2)	Prospective Model (trait emotional intelligence (W1), coping flexibility (W2), and depression (W2) on IGD tendency (W2)) (I) 0.29***	(I) S ( +)	Good (14)
108	Yang et al., 2021a, 2021b	China	244 (70%)	18 – 22 (19.88)	IGD	IAT (29.59 (13.07))	(I) grade point average (T1) (II) IGD (T1)	CL (social cynicism, IGD, and grade point average, after controlling age and gender at T1) (I) -0.17** (II) 0.62***	(I) S (-) (II) L ( +)	Good (14)
109	Yuan et al., 2021	China	341 (75.7%)	21.24 (2.72)	IGD	IGD Questionnaire (Petry et al., 2014) (1.30 (1.98))	(I) Depression symptoms (T1)	Mediation model (Age, gender, depression (T1), FoMO (T2), problematic smartphone use (T3)) (I) 0.31***	(I) M ( +)	Fair (3, 13, 14)
110	Zhang et al., 2019	China	469 (58%)	18 – 27, 19.29 (1.10)	IGD	DSM-5 IGD scale (1.445 (1.968))	(I) Purpose in life (W1) (II) IGD symptoms (W1)	CL (W1, W2: Social support, Purpose in life, IGD symptoms) (I) − 0.173*** (II) 0.365***	(I) S (-) (II) M ( +)	Good (14)
Cross-sectional studies										
111	Borges et al., 2019	Mexico	7022 (55%)	72.4% 18 – 19	IGD	Instrument based on the nine symptoms described in the DSM-5 and formulated by Petry et al. (2015) (5.2% DSM-5 IGD (positive to five or more criteria))	(I) Lifetime psychological (II) Lifetime medical treatment (III) Lifetime any treatment (IV) Severe impairment – home (V) Severe impairment – work/school (VI) Severe impairment – relationships (VII) Severe impairment – social (VIII) Severe impairment – total	LogR (controlling for sex and age group, Lifetime psychological, Lifetime medical treatment, Lifetime any treatment, 12-month treatment, Severe impairment – home, Severe impairment – work/school, Severe impairment – relationships, Severe impairment – social, Severe impairment – total) (I) 1.9* [1.4–2.4] (II) 1.8* [1.1–3.0] (III) 1.8* [1.4–2.4] (IV) 2.1* [1.1–3.8] (V) 2.6* [1.7–4.1] (VI) 1.8* [1.1–2.8] (VII) 1.9* [1.3–3.0] (VIII) 2.4* [1.7–3.3]	(I) S ( +) (II) S ( +) (III) S ( +) (IV) M ( +) (V) M ( +) (VI) S ( +) (VII) S ( +) (VIII) M ( +)	Good (14)
112	Kim & Kim, 2017	Korea	179 (39.1%)	19 – 29, 75.4% 19 – 24	Excessive online game usage	Items based in Young (1998b) and Chin (1998) (2.252 (0.832))	(I) Escaping from loneliness (II) Expanding online bridging social capital (III) Strengthening offline bonding social capital	SEM (Escaping from loneliness, Expanding online bridging social capital, Strengthening offline bonding social capital) (I) 0.44*** (II) 0.221* (III) 0.185*	(I) M ( +) (II) S ( +) (III) S ( +)	Fair (2, 14)
113	Li et al., 2016	China	654 (54%)	18—22 (20.29 (1.39))	Online game addiction	OGCAS (22.92 (9.22); 4.7% (≥ 32, and CIASa ≥ 5))	(I) Avoidant Coping Styles	SEM (Avoidant Coping Styles, stressful life events, neuroticism, stressful live events*neuroticism, Avoidant Coping Styles*neuroticism, controlling for relevant variables (i.e., gender and college year)) (I) 0.199***	(I) S ( +)	Fair (2, 14)
114	Li et al., 2021	China	508 (24%)	18.54 (0.86)	Internet gaming addiction	GAS (2.99 (0.93)	(I) Actual-ideal self-discrepancy (II) Avatar identification (III) Locus of control	SEM (avatar identification, actual-ideal self-discrepancy, locus of control, locus of control*actual-ideal self-discrepancy, locus of control*avatar identification) (I) 0.15** (II) 0.24*** (III) 0.37***	(I) S ( +) (II) S ( +) (III) M ( +)	Good (14)
115	Mills & Allen, 2020	USA	487 (50.3%)	18 – 40, 19.50 (1.90)	IGD	IGD Scale (0.65 (0.77))	(I) Gender (male) (II) General motivation (III) Introjected (IV) Amotivation (V) Self-control	SEM (Gender, Self-Control, Need Frustration, Need Satisfaction, Weekly Playtime, gaming motivations (general, intrinsec, identified, introjected, amotivation, external)) (I) NR (II) 0.59* (III) 0.36* (IV) 0.12* (V) -0.20*	(I) NR (II) L ( +) (III) M ( +) (IV) S ( +) (V) S (-)	Fair (2, 14)
Problematic internet pornography use (PIPU)										
Prospective cohort studies										
116	Grubbs et al., 2018	USA	1507 (34.5%)	19.3 (2.2)	Perceived addiction to internet pornography	CPUI‐9 (1.7 (0.9))	(I) Gender (being male) (II) Access efforts (T1) (III) Perceived compulsivity (T1) (IV) Emotional distress (T1)	MR (personality variables, moral disapproval, religiousness, pornography use and male gender) (I) 0.14* (II) 0.18** (III) 0.30*** (IV) 0.29***	(I) S ( +) (II) S ( +) (III) M ( +) (IV) S ( +)	Fair (2, 14)
Cross-sectional studies										
117	Chen et al., 2018	China	808 (42%)	17 – 22, 18.54 (0.75)	Problematic Pornography Use	PIPUS (7.13 (8.48))	(I) Age (II) Gender (being male) (III) Online sexual activities (IV) Third-person effect	Bootstrapping (Age, gender, sexual sensation seeking, online sexual activities, third-person effect, online sexual activities * third-person effect) (I) − 0.05* (II) − 0.04*** (III) 0.50*** (IV) 0.36***	(I) VS (-) (II) VS ( +) (III) L ( +) (IV) M ( +)	Good (14)

*AOR* adjusted odds ratio; *h* hour; *IGD* Internet gaming disorder; *M* Average; min: minutes; *NR* not reported; *OR* odds ratio; *SD* Standard deviation; *USA* United states of America

^a^Assessment tools: IAT: Internet Addiction Test (Young, 1998a); SAS: Smartphone Addiction Scale (Kwon et al., 2013); SAS-SV: Smartphone Addiction Scale Short Version (Kwon et al., 2013); SAS-C: SAS for college students (Su et al., 2014); MPAI: Mobile Phone Addiction Index Scale (Leung, 2008); PUMPS: Problematic use of mobile phones scale (Merlo et al., 2013); MPPUS: Mobile Phone Problem Use Scale (Bianchi & Phillips, 2005); PCPUQ: Problematic Cellular Phone Use Questionnaire (Yen et al, 2009); SPAI: Smartphone Addiction Inventory Scale (Lin et al., 2014); MPATS: Mobile Phone Addiction Tendency Scale (Xiong et al., 2012); MRCPAS: Manolis/Roberts Cell-Phone Addiction Scale (Roberts et al., 2014); PMPUQ: Problematic Mobile Phone Use Questionnaire (Billieux et al., 2008); PMPUQ-SV: Problematic Mobile Phone Use Questionnaire (Lopez-Fernandez et al., 2018); SDQ: Smartphone Dependence Questionnaire (Salehan & Negahban, 2013); CPDQ: Cell Phone Dependency Questionnaire (Toda et al., 2004); MPAS: Smart Mobile Phone Addiction Scale (Hong et al., 2012); SAPS: Smartphone Addiction Proneness Scale (Kim et al., 2014); BFAS: Bergen Facebook Addiction Scale (Andreassen et al., 2012); BSNAS: Bergen Social Networking Addiction Scale (Andreassen et al., 2012); BSMAS: Bergen Social Media Addiction Scale (Andreassen et al., 2016); FIQ: Facebook Intrusion Questionnaire (Elphinston & Noller, 2011); GPIUS-2: Generalized Problematic Internet Use Scale-2 (Caplan, 2010); CIUS: Compulsive Internet Use Scale (Meerkerk, 2007); CERI: Cuestionario de Experiencias Relacionadas con Internet (Beranuy et al., 2009); SMUQ: Social Media Use Questionnaire (Xanidis & Brignell, 2016); OGCAS: Online Game Cognitive Addiction Scale (Li et al., 2008); GAS: Game Addiction Scale (Lemmens et al., 2015); CPUI‐9: Cyber Pornography Use Inventory‐9 (Short et al., 2012)

^b^Statistical model: R: Regression analysis; BLogR: Binary logistic regression; CL: Cross-lagged model; HR: Hierarchical regression; HLR: Hierarchical linear regression; HMLR: Hierarchical multiple linear regression; HMR: Hierarchical multiple regression; LRA: Linear regression analysis; LogR: Logistic regression; MLR: Multiple linear regression; MR: Multiple regression; MLogR: Multivariant logistical regression; SEM: Structural equation model; PLS-SEM: Partial least square structural equation modeling; MIMIC: Multiple indicators multiple causes model; BA: Bayesian approach; PM: Path model. *p < 0.05; **p < 0.01; ***p > 0.001

^c^Direction of the association: + : positive association; –: negative association. Effect size: VS: very small; S: small; M: moderate; L: large. Interpretation: β: VS > 0 to < 0.1, S ≥ 0.1 to < 0.3, M ≥ 0.3 to < 0.5 and L ≥ 0.5 (Cohen, 2013; Ferguson, 2016); OR: VS > 0 to < 1.5, S ≥ 1.5 to < 2, M ≥ 2 to < 3 and L ≥ 3 (Sullivan & Feinn, 2012); R2: VS > 0 to < 0,02, S ≥ 0,02 to < 0.13, M ≥ 0.13 to < 0.26 and L ≥ 0.26 (Dominguez-Lara, 2017)

^d^QA: Quality assessment: Numbers represent unmet quality criteria from Table 3 (NHLBI et al., 2014)

### Quality assessment

The "quality assessment tool for observational cohort and cross-sectional studies" was used (National Heart, Lung, and Blood Institute, 2014). 50.4% of articles (*n* = 59) were assessed by the first and second authors. After obtaining an adequate interjudge agreement (98.9%), the first author completed the quality assessment.

Articles are classified as "good", "fair" or "poor" quality based on an overall judgment based on 14 criteria (Table 3). Four are not applicable to cross-sectional studies (6, 7, 10 and 13). All meet the 4th and 9th criteria (university student, over 17 years old and measure problematic behaviour by validated questionnaire). All meet the 12th criterion. They were filled in anonymously. Compliance with the 2nd criterion (demographics, location and time period when they were obtained) is considered to be of good quality. For the 8th criterion, a dichotomized measurement of the independent variable is considered to be of good quality if the cut-off point used is validated. For the 14th criterion, a model controlling the variables of sex (if not stratified), age (if not in the range ± 1 year), and a third confounding factor (for example, socioeconomic status or academic performance) is considered sufficient. 38.5% of the articles have not reported participation rates, preventing the 3rd criterion from being assessed.

**Table 3:** Checklist criteria, from the U.S. Department of Health & Human Services

1	Research objective
2	Study population
3	Participation rate ≥ 50%
4	Recruitment
5	Sample size
6	Exposure before outcome
7	Timeframe
8	Levels of exposures
9	Exposure measurement
10	Exposure assessment in time
11	Outcome measurement
12	Blindness
13	Loss to follow-up ≤ 20%
14	Confounding

### Data analysis

In Objectives 1, 2 and 3, a descriptive analysis of the 117 articles included was carried out (See Table 2). Objective 4 analysed all 83 studies, examining 17 predictive factors included in the review.

## Results

The database search identified 117 studies that examined risk and protective factors for problem *behaviours online* (see Table 2).

In the quality assessment, 56 articles (47.9%) were of good quality, 30 (52.1%) were medium, and none displayed poor quality. The most frequent limitations were lack of complete definition of the population (2nd criterion), non-contemplation of different levels of exposure of potential predictive factors (8th criterion), and no introduction of confounding variables in predictive models (14th criterion).

The articles included have been divided into four groups based on specific online behaviour: PSU (*n* = 67), PSMU (*n* = 39), IGD (*n* = 9), y PIPU (*n* = 2). These five constructs are treated separately because research suggests that their prevalence rates and risk factors appear to be different from each other (Billieux, 2012; Kuss et al., 2021).

### Problematic smartphone use

A total of 67 studies have analysed the predictive factors for PSU in college students.

#### Description of studies

The design was longitudinal in 5 studies (7.5%) (Cui et al., 2021; Elhai et al., 2018b; Rozgonjuk et al., 2019; Yuan et al., 2021; Zhang et al., 2021) and in 62 it was cross-sectional (92.5%). 75% were from Asia (*n* = 50) and 16.4% from North America (USA) (*n* = 11), and a smaller number were from Europe (*n* = 4) and South America (*n* = 2, 3%). 29.8% of the studies were published between 2013—2017, and 70.2% from 2018. The samples ranged from 191 (Roberts and Pirog III, 2013) to 2367 students (Alosaimi et al., 2016), with 82.1% below 1000 and a mean of 617.3 (SD = 392.8).

The terms ‘smart/mobile/cell phone addiction’ (*n* = 31, 46.3%), ‘problematic smart/mobile phone use’ (*n* = 30, 44.8%), ‘smartphone use disorder’ (*n *= 2, 3%), ‘mobile phone dependence’ (*n* = 2, 3%), ‘excessive smartphone use’ (*n* = 1 1.5, 1.5%) and ‘smartphone dependency’ (*n *= 1%) were used.

17 assessment instruments were identified (see Table 4). High scores indicated a higher degree of PSU. The most widely used was the Smartphone Addiction Scale (SAS) (Kwon et al., 2013) (*n* = 24, 35.8%). Different versions of this instrument have been used. 14 used the short version (range: 10—60) with means between 20.96 (SD = 7.56) in Turkey (Gökçearslan et al., 2016) and 41.35 (SD = 35.95) in Italy (De Pasquale et al., 2019). Eight used the standard version (range: 33—198) with means between 68.46 (SD = 24.95) in South Korea (Choi et al., 2015) and 98.00 (SD = 26.73) in Brazil (Laurence et al., 2020), and 2 used the undergraduate version (Su et al., 2014) (range: 1—5) with means between 2.85 (SD = 0.55) (Lin et al., 2021) and 2.88 (SD = 0.566) (He et al., 2020) both in China. Ten studies used the ‘Mobile Phone Addiction Index Scale’ (Leung, 2008) (range = 1—5) with averages between 1.99 (SD = 0.58) (Zhang et al., 2020a) and 2.78 (SD = 0.72) (Yang et al., 2020b), both in China.

**Table 4:** Assessment tools

Assessment tools	*n*	M (SD)	Prevalence % (cut-off point)
Problematic Smartphone use (PSU)			
*Smartphone Addiction Scale* (SAS) (Kwon et al., 2013) (range: 33—198)	8		
Choi et al., 2015		68.46 (24.95)	NR
Enez Darcin et al., 2016		87.6 (26.45)	NR
Elhai et al., 2018c		93.47 (25.30)	NR
Elhai et al., 2018a		93.53 (25.38)	NR
Erdem & Uzun, 2020		78.93 (23.21)	NR
Laurence et al., 2020		98.00 (26.73)	NR
Rozgonjuk & Elhai, 2021		93.47 (25.30)	NR
Wolniewicz et al., 2020		91.52 (23.95)	NR
*Smartphone Addiction Scale* Short Version (SAS-SV) (Kwon et al., 2013) (range: 10—60)	14		
Abbasi et al., 2021		NR	NR
Coban & Gundogmus, 2019		NR	46.9 (≥ 31 M, ≥ 33 F)
De Pasquale et al., 2019		41.35 (35.95)	NR
Elhai et al., 2018b		26.31 (10.35)	NR
Elhai et al., 2020b		34.92 (11.39)	NR
Elhai et al., 2020a		27.41 (9.41)	NR
Elhai et al., 2020c		37.36 (9.54)	NR
Forster et al., 2021		NR	24.32 (≥ 32)
Gökçearslan et al., 2016		20.96 (7.56)	NR
Gündoğmuş et al., 2021		NR	48.6 (≥ 31 M, ≥ 33 F)
Kuru & Celenk, 2021		29.50 (11.34)	NR
Rozgonjuk et al., 2019		26.31 (10.35)	NR
Yuan et al., 2021		35.23 (10.58)	NR
Yuchang et al., 2017		23.75 (7.47)	27.92 (≥ 31 M, ≥ 33 F)
SAS for college students (SAS-C) (Su et al., 2014) (range: 1—5)	2		
He et al., 2020		2.88 (0.57)	NR
Lin et al., 2021		2.85 (0.55)	NR
Mobile Phone Addiction Index Scale (MPAI) (Leung, 2008) (range: 1—5)	10		
Hou et al., 2021		2.7 (0.71)	NR
Lian & You, 2017		2.89 (0.66)	52,9 (scored highest 27%)
Lian, 2018		2.64 (0.67)	NR
Lin & Chiang, 2017		NR	NR
Liu et al., 2020		2.69 (0.70)	NR
Xiao et al., 2021		2.78 (0.72)	NR
Yang et al., 2020a, 2020b		2.63 (0.63)	NR
Zhang et al., 2020b		2.66 (0.58)	NR
Zhang et al., 2020a		1.99 (0.58)	NR
Zhu et al., 2019		2.51 (0.64)	NR
Problematic use of mobile phones scale (PUMPS) (Merlo et al., 2013) (range: 20—100)	5		
Alosaimi et al., 2016		60.8	NR
Cebi et al., 2019		59.87 (16.92)	NR
Jiang & Zhao, 2016		41.07 (9.94)	NR
Jiang & Zhao, 2017		41.07 (9.94)	NR
Jiang & Shi, 2016		NR	8.99 (NR)
Mobile Phone Problem Use Scale (MPPUS) (Bianchi & Phillips, 2005) (range: 19—190)	2		
Pourrazavi et al., 2014		NR	25.5 (NR)
Takao, 2014		103.7 (38.88)	NR
Problematic Cellular Phone Use Questionnaire (PCPUQ) (Yen et al, 2009)	1		
Long et al., 2016		NR	21.3 (≥ 4 of the first 7 questions and any of the last 5 questions)
Smartphone Addiction Inventory Scale (SPAI) (Lin et al., 2014) (Ranges: 0—26; 5 – 95; 24 – 94; 26 – 104)	5		
Arpaci & Kocadag Unver, 2020		NR (range: 26—104)	NR
Bian and Leung, 2014		48.48 (12.75) (range: 5—95)	NR
Giordano et al., 2019		37.07 (10.60) (range: 24—96)	NR
Khoury et al., 2019		NR (range: 0—26)	43.85 (≥ 7)
Matar Boumosleh & Jaalouk, 2017		55.37 (15.04) (range: 26–104)	NR
Mobile Phone Addiction Tendency Scale (MPATS) (Xiong et al., 2012) (Range: 1 – 5; 16—80)	6		
Cui et al., 2021		37.08 (13.62) (Range: 16—80)	NR
Sun et al., 2021		2.634 (0.70) (Range: 1 – 5)	NR
Yang et al., 2019		42.81 (10.63) (Range: 16—80)	NR
Yang et al., 2021a, 2021b		NR (Range: 16—80)	78.29 (32–56), 8.06 (≥ 57)
You et al., 2019		2.73 (0.69) (Range: 1 – 5)	NR
Zhang et al., 2021		2.60 (0.60) (Range: 1 – 5)	NR
Mobile phone technology addiction scale (Ehrenberg et al., 2008) (Range: 1—7)	1		
Roberts & Pirog III, 2013		5.093 (1.272)	NR
Manolis/Roberts Cell-Phone Addiction Scale (MRCPAS) (Roberts et al., 2014)	1		
Roberts et al., 2015		NR	NR
Problematic Mobile Phone Use Questionnaire (PMPUQ; Billieux et al., 2008) and Mobile Phone Addiction Index (MPAI; Leung, 2008) (Range: 1—5)	1		
Liu et al., 2021		2.72 (0.83)	NR
‘Problematic Mobile Phone Use Questionnaire’ (PMPUQ-SV) (Lopez-Fernandez et al., 2018) (Range: 5—20Addictive mobile phone use; 5—16 Antisocial mobile phone use; 5—19 Dangerous mobile phone use)	1		
Canale et al., 2021		12.88 (3.03); 9.78 (2.35); 7.94 (2.95)	NR
Smartphone Addiction Proneness Scale (Kim et al., 2014) (Range: 15—75)	4		
Kim et al., 2017		NR	NR
Kim & Koh, 2018		33.45 (7.67)	NR
Kim et al., 2019		NR	36 (40–43), 30.2 (≥ 44)
Rozgonjuk et al., 2018		33.58(12.12)	NR
Smartphone Dependence Questionnaire (SDQ) (Salehan & Negahban, 2013) (Range: 7—49)	1		
Koç & Turan, 2021		2468 (0.777)	NR
Salehan & Negahban, 2013		NR	NR
‘Cell Phone Dependency Questionnaire’ (CPDQ, Toda et al., 2004)	1		
Alavi et al., 2020		NR	NR
‘Smart Mobile Phone Addiction Scale’ (MPAS) (Hong et al., 2012) (Range: 1—6)	1		
Kuang-Tsan & Fu-Yuan, 2017		2.05	NR
de Zhitomirsky-Geffet & Blau (2016) (Range: 1—5)	1		
Handa & Ahuja, 2020		2.98 (0.58)	NR
Modified from Hong, Chiu, and Huang (2012) (Range: 11–59)	1		
Hong et al., 2021		33.82 (10.23)	NR
Problematic social media use (PSMU)			
Bergen Facebook Addiction Scale (BFAS; Andreassen et al., 2012) (Range: 1 – 5; 6 – 30; 18 – 90)	13		
Balcerowska et al., 2019		12.88 (4.93) (range: 6—30)	NR
Brailovskaia & Margraf, 2017		9.77 (3.86) (range: 6—30)	NR
Brailovskaia et al., 2018		8.98 (3.64) (range: 6—30)	NR
Casale & Fioravanti, 2018		1.67 (0.64) (range: 1—5)	NR
Dempsey et al., 2019		11.33 (5.06) (range: 6—30)	NR
Foroughi et al., 2021^a^		NR	NR
Punyanunt-Carter et al., 2018^b^		NR (range: 6—30)	36.9 (≥ 18)
Satici & Uysal, 2015		32.43 (14.83) (range: 18—90)	NR
Sayeed et al., 2020		NR (range: 6—30)	NR
Sheldon et al., 2021^a, c^		1.91 (0.73), 2.26 (0.93), 2.08 (0.94) (range: 1—5)	NR
Uysal, 2015		30.09 (10.21) (range: 18—90)	NR
Lee, 2019		NR	NR
Xie & Karan, 2019		3.15 (0.71) (range: 1—5)	NR
Bergen Social Networking Addiction Scale (Andreassen et al., 2012) (Range: 6 – 30)	1		
Siah et al., 2021		NR	NR
Bergen Social Media Addiction Scale (BSMAS; Andreassen et al., 2016) (Range: 1- 5; 6—30)	4		
Casale et al., 2018		11.96 (4.99) (range: 6—30)	NR
Chung et al., 2019		16.74 (4.16) (range: 6—30)	NR
Varchetta et al., 2020		2.21 (0.81) (range: 1—5)	NR
Yu & Chen, 2020		NR	NR
Facebook Intrusion Questionnaire (FIQ; Elphinston & Noller, 2011) (Range: 10—50)	2		
Hou et al., 2017b		19.61 (6.15)	NR
Hou et al., 2019		19.43 (7.14)	NR
Social Network Addiction Questionnaire (Escurra & Salas, 2014) (Rango: 1—5)	1		
Jasso-Medrano & Lopez-Rosales, 2018		2.33 (0.71)	NR
Excessive WeChat Use scale (Hou et al., 2017a, 2017b) (Range: 1—5)	2		
Gao et al., 2021		2.54 (0.76)	NR
Hou et al., 2017a, 2017b		15.00	8.2 (21.4–27.7), 6.6 (> 27.7)
Internet Addiction Test (IAT) (Young, 1998a)	4		
Hong et al., 2014^d^ (Ranges: Withdrawal 3—13; Tolerance: 3 – 18; Life problems 3—17; Substitute satisfaction 3—18)		5.55 (2.90); 9.26 (3.58); 7.29 (3.29); 8.49 (3.33)	NR
Hong & Chiu, 2016^d^ (Ranges: Withdrawal and tolerance 3—18; Life problems 3—16; Substitute satisfaction 3—18)		6.37 (3.30); 5.93 (2.91); 8.86 (3.66)	NR
Jaradat & Atyeh, 2017 (Range: Withdrawal, Tolerance, Life problems, Substitute satisfaction: 1 – 5; Total: 20—100)		2.63 (1.05); 3. 06 (1.20); 3.12 (0.96); 2.98 (0.10)	62.1 (50–79); 7.9 (≥ 80)
Kircaburun & Griffiths, 2018^f^ (Range: 15—90)		NR	26.5 (38–58), 6.1 (59–73), 0.9 (> 73)
Generalized Problematic Internet Use Scale-2 (GPIUS-2) (Caplan, 2010) (Range: 15—120)	2		
Marino et al., 2016^d^		28.74 (14.12)	NR
Casale & Fioravanti, 2017^e^		33.14 (16.03)	NR
Compulsive Internet Use Scale (CIUS) (Meerkerk, 2007) (Range: 1—5)	1		
Aladwani & Almarzouq, 2016^ g^		2.51 (0.71)	NR
Cuestionario de Experiencias Relacionadas con Internet (CERI) (Beranuy et al., 2009) (Range: 1—5)	1		
Duran, 2015^ h^		1.44 (0.79)	NR
Items adapted from Su and Chan (2017)	1		
Raza et al., 2020		NR	NR
Social media addiction scale (Tutgun-Ünal & Deniz, 2015) (Range: 1—5)	1		
Demircioğlu & Köse, 2020		2.18 (0.70)	NR
Demircioğlu & Göncü Köse, 2021		2.15 (0.70)	NR
Social Media Use Questionnaire (SMUQ) (Xanidis & Brignell, 2016) (Range: 1—5; 9—45)	3		
Kircaburun et al., 2020b		24.10 (6.73) (range: 9—45)	NR
Kircaburun et al., 2020a		15.21 (7.48) (range: 9—45)	NR
Süral et al., 2019		2.71 (0.75) (range: 1—5)	NR
Social networking sites addiction scale (Wang, 2016) (Range: 1—5)	1		
Shan et al., 2021		2.85 (0.70)	NR
Social Media Disorder Scale (Van den Eijnden et al., 2016) (Range: 1—5)	1		
Yu & Luo, 2021		2.80 (2.21)	NR
Internet gaming disorder (IGD)			
Chinese version of the Online Game Cognitive Addiction Scale (OGCAS; Li et al., 2008) (Range: 16—80)	1		
Li et al., 2016		22.92 (9.22)	4.7 (≥ 32, and CIAS^i^ ≥ 5)
Questionnaire ad hoc based in Young (1998b) and Chin (1998) (Range: 1—5)	1		
Kim & Kim, 2017		2.252 (0.832)	NR
Internet Gaming Disorder Questionnaire (Petry et al., 2014) (Range: 0—9)	1		
Yuan et al., 2021		1.30 (1.98)	NR
Instrument based on the nine symptoms described in the DSM-5 and formulated by Petry et al. (2015) (Range: 0—23)	1		
Borges et al., 2019		NR	5.2 (≥ 5)
DSM-5 IGD scale (APA, 2013) (Range: 0—9)	2		
Zhang et al., 2019		1.44 (1.97)	NR
Dang et al., 2019		1.45 (1.97)	NR
Internet Gaming Disorder Scale (Lemmens et al., 2015) (Range: 0—5)	1		
Mills & Allen, 2020		0.65 (0.77)	NR
Internet Addiction Test (IAT; Young, 1998a)^j^ (Range: 20—100)	1		
Yang et al., 2021a, 2021b		29.59 (13.07)	NR
Game Addiction Scale (GAS) (Lemmens et al., 2009) (Range: 0—5)	1		
Li et al., 2021		2.99 (0.93)	NR
Problematic Online pornography Use			
Problematic Pornography Use Scale (Kor et al., 2014) (Range: 0—60)	1		
Chen et al., 2018		7.13 (8.48)	NR
Cyber Pornography Use Inventory‐9 (CPUI‐9) (Short et al., 2012) (Range: 1 – 7)	1		
Grubbs et al., 2018		1.7 (0.9)	NR

^a^The word “Facebook” was replaced with “Instagram”

^b^The word “Facebook” was replaced with social media in general

^c^The word “Facebook” was replaced with “Snapchat”

^d^The word “Internet” was replaced with “Facebook”

^e^The word “Internet” was replaced with “Social Network Sites”

^f^The word “Internet” was replaced with “Instagram”

^g^The word “Internet” was replaced with “Social Media”

^h^The word “Internet” was replaced with “Tuenti”

^i^Chinese version of the Internet Addiction Scale (CIAS; Zhu & Wu, 2004)

^j^ Replacing the words “online” or “Internet” with words such as “play Internet game”

Eleven studies (16.4%) reported the prevalence. Thus, the study by Yang et al. (2021a) using the 'Mobile phone addiction tendency scale (MPATS)', with a total score of 16 to 80 (a higher score indicating a deeper degree of addiction), and dividing the total sample into three groups (from 16 to 31 is classified as "no mobile phone addiction", from 32 to 56 is classified as "possible mobile phone addiction", and those equal to or higher than 57 are classified as "mobile phone addiction"), found that 78.29% were classified as possible addiction and 8.06% as addiction. In contrast, the study by Jiang and Shi (2016), using the ‘Problematic Mobile Phone Use Scale (PMPUS)’ and making a dichotomous division, finds that 8.99% display problematic use. Meanwhile, the study by Gündoğmuş et al. (2021), using 'The Smartphone Addiction Scale-Short Version (SAS-SV)', with a score between 10 and 60, and making a dichotomous division (yes/no) finds 48.6% of participants fall within the "addiction" group (SAS-SV ≥ 31 for boys and SAS-SV ≥ 33 for girls). The study by Long et al. (2016) used the Problematic Cellular Phone Use Questionnaire (PCPUQ), a questionnaire composed of 12 items, the first seven of which asked whether in the previous year the participants had symptoms of problematic CPU, while the last five determined the subjective functional impairment of the participants in the previous year caused by CPU, so that participants who had positive responses to four or more of the first seven questions and those who had positive responses to any of the last five questions were classified as having problematic CPU, and found a prevalence of 21.3%.

#### Predictive factors

From the 67 studies of adequate quality, 10 potential predictive factors of PSU were extracted, classified into two categories (Internet use patterns and psychological variables) (see Table 5).

**Table 5:** Problematic smartphone use predictive factors

	Internet use patterns			Psychological variables						
	Smartphone use	Social media use	No social use (process use)	Negative affectivity	Self-control/regulation	Well-being	Neuroticism/ emotional instability	FoMO	Impulsivity	Other online addictions
*n*	10	6	3	25	7	12	6	8	4	3
Nº de sujetos	7,097	3,724	877	18,694	4,746	7,443	3,591	4,484	1,747	1,219
Abbasi et al., 2021		+ +								
Alavi et al., 2020				+ , + + + + ^a^						
Alosaimi et al., 2016	+ +									
Canale et al., 2021				+ , NS^a^		NS	NS		+ + , + + + ^a^	
Cebi et al., 2019					–, –-^a^					
Choi et al., 2015				+ +						+ +
Coban & Gundogmus, 2019		+ + +								
Cui et al., 2021				+ +						
De Pasquale et al., 2019							–^b^			
Elhai et al., 2020a			+ +					+ + + +		
Elhai et al., 2018a	+ + +			+ + +				+ + +		
Elhai et al., 2018b						–				
Elhai et al., 2018c	+ +									
Elhai et al., 2020b	+ +							+ + + +		
Elhai et al., 2020c				+				+ +		
Enez Darcin et al., 2016				+ + +						
Erdem & Uzun, 2020	+ +						+ +			
Forster et al., 2021				+ , + + , NS^a^						
Gökçearslan et al., 2016	+ + + +				–	NS				
Handa & Ahuja, 2020								+ + +		
He et al., 2020				+ +						
Hong et al., 2021	+ +									
Hou et al., 2021				+ + +						
Jiang & Shi, 2016					-	-				
Khoury et al., 2019				+ + + +					+	+ + + +
Kim & Koh, 2018				+ +		–				
Kim et al., 2017				+ + +						
Kim et al., 2019				+ + , + + + ^a^						
Koç & Turan, 2021		+ + + +				–				
Kuang-Tsan & Fu-Yuan, 2017				+ + , + + + , NS^a^						
Kuru & Celenk, 2021				+ +						
Lian & You, 2017						–, + +				
Lian, 2018						–, NS				
Lin & Chiang, 2017		+ , + + ^a^								
Lin et al., 2021	+ + +							+ +		
Liu et al., 2020				+ + +			+ +			
Liu et al., 2021				+ +						
Long et al., 2016				+						
Matar Boumosleh & Jaalouk, 2017	+ + , NS^a^			+ +						
Pourrazavi et al., 2014					-, –^a^					
Roberts & Pirog III, 2013									+ +	
Roberts et al., 2015							+ +		+ +	
Rozgonjuk et al., 2018		+ + + +								
Rozgonjuk et al., 2019			+ + +							
Rozgonjuk & Elhai, 2021			+ +							
Salehan & Negahban, 2013		+ + + +								
Sun et al., 2021						–				
Takao, 2014							–^b^			
Wolniewicz et al., 2020	+ +							+ + + +		
Xiao et al., 2021				+ +						
Yang et al., 2019					––					
Yang et al., 2020a, 2020b				+ +	–					
You et al., 2019				NS		NS				
Yuan et al., 2021				+ +				+ +		+ +
Yuchang et al., 2017						–-				
Zhang et al., 2020a				+ +						
Zhang et al., 2020b						–				
Zhang et al., 2021					–-					

*n*: sample size (no. of studies); + : positive association (risk factor); –: negative association (protective factor). Effect size: ±  = very small (VS); +  ±—= small (S); +  +  ± – = medium (M); +  +  +  ± –- = large (L). Interpretation: R^2^: VS > 0 to < 0.1, S ≥ 0.1 to < 0.3, M ≥ 0.3 to < 0.5 and L ≥ 0.5 (Cohen, 2013; Ferguson, 2016); OR: VS > 0 to < 1.5, S ≥ 1.5 to < 2, M ≥ 2 to < 3 and L ≥ 3 (Sullivan & Feinn, 2012); R2: VS > 0 to < 0,02, S ≥ 0,02 to < 0.13, M ≥ 0.13 to < 0.26 and L ≥ 0.26 (Dominguez-Lara, 2017). NS = not significant association; NR = not reported

^a^ More than one effect size corresponding to more than one variable as a measure of potential predictor. ^b^ Emotional stability

##### Internet use patterns

*Smartphone use (time, frequency).* Out of 10 studies, all reported that smartphone use was a potential risk factor for PSU (Alosaimi et al., 2016; Elhai et al., 2018a; Elhai et al., 2018c; Elhai et al., 2020b; Erdem & Uzun, 2020; Gökçearslan et al., 2016; Hong et al., 2021; Lin et al., 2021; Wolniewicz et al., 2020). Seven showed a small effect size (70%), two a moderate size (20%) and one a large effect size (10%). The study by Matar Boumosleh and Jaalouk (2017) found a significant effect of excessive mobile phone use (> 5 h per day) but not of daily usage time.

*Social media use.* Out of 6 studies, all reported that social media use was a potential risk factor for PSU (Abbasi et al., 2021; Coban & Gundogmus, 2019; Koç & Turan, 2021; Lin & Chiang, 2017; Rozgonjuk et al., 2018; Salehan & Negahban, 2013). One showed a small effect size (16.7%), one a moderate size (16.7%), three a large effect size (50%) and one showed a mixed effect (16.7%).

*Non social use (process use).* Out of 3 studies, all reported that non-social use (process use) was a potential risk factor for PSU (Elhai et al., 2020a; Rozgonjuk & Elhai, 2021; Rozgonjuk et al., 2019). Two showed a small effect size (66.6%) and one moderate (33.3%).

##### Psychological variables

*Negative affectivity (depression, anxiety, stress, boredom proneness, rumination, suicidal ideation)*. Out of 25 studies, 24 reported that negative affectivity was a potential risk factor for PSU (Alavi et al., 2020; Canale et al., 2021; Choi et al., 2015; Cui et al., 2021; Elhai et al., 2018a; Elhai et al., 2020c; Enez Darcin et al., 2016; Forster et al., 2021; He et al., 2020; Hou et al., 2021; Khoury et al., 2019; Kim & Koh, 2018; Kim et al., 2017; Kim et al., 2019; Kuang-Tsan & Fu-Yuan, 2017; Kuru & Celenk, 2021; Liu et al., 2020; Liu et al., 2021; Long et al., 2016; Matar Boumosleh & Jaalouk, 2017; Xiao et al., 2021; Yang et al., 2020a, 2020b; You et al., 2019; Yuan et al., 2021; Zhang et al., 2020a). Two showed a very small effect size (8.3%), 11 small (45.8%), five moderate (20.8%), one large (4.2%) and five mixed (20.8%).

Specifically, the studies found that depression (Alavi et al., 2020; Choi et al., 2015; Cui et al., 2021; Forster et al., 2021; Kim et al., 2017; Matar Boumosleh & Jaalouk, 2017; Yang et al., 2020a, 2020b; Yuan et al., 2021; Zhang et al., 2020a), anxiety (Alavi et al., 2020; Choi et al., 2015; Hou et al., 2021; Khoury et al., 2019; Kim & Koh, 2018; Kuru & Celenk, 2021; Matar Boumosleh & Jaalouk, 2017), social anxiety (Canale et al., 2021; Enez Darcin et al., 2016; Xiao et al., 2021; You et al., 2019), depression/anxiety and suicidal ideation (Kim et al., 2019), stress (Forster et al., 2021; He et al., 2020; Kim et al., 2019; Kuang-Tsan & Fu-Yuan, 2017; Liu et al., 2020; Long et al., 2016), rumination (Elhai et al., 2020c; Liu et al., 2021) and boredom proneness (Elhai et al., 2018a; Yang et al., 2020a, b; Zhang et al., 2021) were risk factors.

*Self-control/regulation.* Out of 7 studies, all reported that self-control/self-regulation was a potential protective factor against PSU (Cebi et al., 2019; Gökçearslan et al., 2016; Jiang & Shi, 2016; Pourrazavi et al., 2014; Yang et al., 2019, 2020a, 2020b; Zhang et al., 2021). One showed a very small effect size (14.3%), two small (28.6%), one moderate (14.3%), one large (14.3%) and two mixed (28.6%).

*Well-being (self-efficacy, tolerance to distress, self-esteem, vitality, interpersonal adaptation, *etc.*).* Out of 13 studies, 9 reported that well-being was a potential protective factor with respect to PSU (Elhai et al., 2018b; Jiang & Shi, 2016; Kim & Koh, 2018; Koç & Turan, 2021; Lian, 2018; Lian & You, 2017; Sun et al., 2021; Yuchang et al., 2017; Zhang et al., 2020b). One showed a very small effect size (11%), five small (55.6%), two moderate (22.2%) and two mixed (22.2%).

Specifically, the studies found self-efficacy (Jiang & Shi, 2016), social self-efficacy (Sun et al., 2021), distress tolerance (Elhai et al., 2018b), self-esteem (Jiang & Shi, 2016; Kim & Koh, 2018; Koç & Turan, 2021; Yuchang et al., 2017), relationship virtues (Lian, 2018; Lian & You, 2017), interpersonal adaptation (Zhang et al., 2020b) and vitality (Lian & You, 2017),

*Neuroticism/emotional instability.* Out of 6 studies, three found neuroticism to be a potential risk factor for PSU (Erdem & Uzun, 2020; Liu et al., 2020; Roberts et al., 2015), while two found that emotional stability was a protective factor (De Pasquale et al., 2019; Takao, 2014). These five studies reported a small effect size. 

*Fear of Missing Out (FOMO).* Out of 8 studies, all reported that FOMO was a potential risk factor for PSU (Elhai et al., 2018a; Elhai et al., 2020a, 2020b, 2020c; Handa & Ahuja, 2020; Lin et al., 2021; Wolniewicz et al., 2020; Yuan et al., 2021). Three of them reported a small effect size (37.5%), two a moderate one (25%) and three a large effect size (37.5%). *Impulsivity.* Out of 4 studies, all reported impulsivity as a potential risk factor for PSU (Canale et al., 2021; Khoury et al., 2019; Roberts & Pirog III, 2013; Roberts et al., 2015). One showed a small effect size (25%), two a small one (50%) and one a mixed effect size (25%).

*Other online addictions.* Out of 3 studies, all reported that other cyber addictions were potential risk factors for PSU, namely internet addiction (Choi et al., 2015), Facebook addiction (Khoury et al., 2019), and IGD (Yuan et al., 2021). The reported effect sizes were small in two studies (66.6%) and moderate in one (33.3%).

### Problematic social media use

A total of 39 studies have analysed the predictive factors for PSMU in college students.

#### Description of studies

The design was longitudinal in two studies (5.1%) (Brailovskaia & Margraf, 2017; Brailovskaia et al., 2018) and transverse in 37 (94.9%). 64.1% were from Asia (*n* = 25) and 23% from Europe (*n* = 9), and a smaller number were from North America (USA) (*n* = 4, 10.3%) and Central America (Mexico) (*n* = 1, 2.6%). 30.8% of the studies were published between 2014—2017, and 69.2% from 2018. The samples ranged from 122 (Brailovskaia et al., 2018) to 1245 students (Hou et al., 2017a), with 94.9% below 1000 and a mean of 433.92 (SD = 240.8).

The following terms were used**:** ‘social media addiction’ (*n* = 8, 20.5%), ‘social networking sites addiction’ (*n* = 4, 10.2%), ‘problematic social media use’ (*n* = 4, 10.2%), ‘poblematic social networking sites use’ (*n* = 3, 7.7%) and ‘compulsive social media use’ (*n* = 1, 2.5%). Others studied the problematic use of certain social networks: ‘Facebook addiction’ (*n* = 7, 17.9%), ‘problematic Facebook use’ (*n* = 4, 10.2%), ‘Facebook addiction disorder’ (*n* = 2, 5.1%), ‘intensive Facebook usage’ (*n* = 1, 2.5%), ‘WeChat excessive use’ (*n* = 2, 5.1%), ‘Tuenti addiction’ (*n* = 1, 2.5%), ‘Instagram addiction’ (*n* = 3, 7.7%) y ‘Snapchat addiction’ (*n* = 1, 2.5%).

16 assessment instruments were identified (see Table 4). High scores indicated a higher degree of PSMU. The most widely used was the Bergen Addiction Scale in its different ranges (*n* = 18, 46.1%). Among them, 13 studies used the Bergen Facebook Addiction Scale (BFAS; Andreassen et al., 2012) with different ranges (1 – 5; 6 – 30; 18 – 90). Out of the 6 studies using the 6—30 range, the means went from 8.98 (SD = 3.64) (Brailovskaia et al., 2018) to 12.88 (4.93) (Balcerowska et al., 2019). The study by Siah et al. (2021) used the Bergen Social Networking Addiction Scale (Andreassen et al., 2012) and four studies used its most up-to-date version, the Bergen Social Media Addiction Scale (BSMAS; Andreassen et al., 2016). Of these latter studies, among those who used a range of 6—30, scores went from 11.96 (SD = 4.99) (Casale et al., 2018) to 16.74 (SD = 4.16) (Chung et al., 2019). On the other hand, 8 studies used specific Generalized PIU instruments such as the Internet Addiction Test (IAT) (Young, 1998a), Generalized Problematic Internet Use Scale-2 (GPIUS-2 2009) (Caplan, 2010), Compulsive Internet Use Scale (CIUS) (Meerkerk, 2007), and the Internet Experience Questionnaire (CERI) (Beranuy et al., 2009).

Four studies (10.2%) reported on the prevalence. So, the study by Punyanunt-Carter et al.  (2018) using the Bergen Facebook Addiction Scale (BFAS; Andreassen et al., 2012), with a total score of 6 to 30, and using a cut-off point of ≥ 18, found that 36.9% had PSMU. Hou et al. (2017a) Using the ‘Excessive WeChat Use scale’ and using two cut-off points (21.4–27.7, > 27.7), reported that 8.2% had ‘excessive use” and 6.6% “serious excessive use”. Kircaburun and Griffiths (2018), using the Internet Addiction Test (IAT) (Young, 1998a) (range: 20–100) and three cut-off points (38–58, 59–73, > 73), found that 26.5% had mild addiction, 6.1% moderate addiction, and 0.9% severe addiction. Jaradat and Atyeh (2017), using the Internet Addiction Test (IAT) (Young, 1998a) (range: 20–100) and using two cut-off points, (50–79, ≥ 80), reported that 62.1% were in the alert group, and 7.9% displayed levels of addiction.

#### Predictive factors

5 potential predictive factors of PSMU were extracted from the 39 studies of adequate quality, and they were classified into two categories (Internet use patterns and psychological variables) (see Table 6).

**Table 6:** Problematic social media use predictive factors

	Internet use patterns		Psychological variables		
	Social media use (time, frequency)	Social use	Negative affect (depression, anxiety, social media communication apprehension, rumination)	Well-being (flourishing, life satisfaction, social safeness, relationship satisfaction, trait emotional inteligence, self-confidence, self-esteem, vitality, self-liking, psychological capital, psychological resilience)	FoMO
n	9	6	10	11	4
Nº de sujetos	3,300	3,117	4,656	5,361	1,513
Aladwani & Almarzouq, 2016				–	
Casale et al., 2018					+ + +
Chung et al., 2019	+ +				
Demircioğlu & Göncü Köse, 2021				–	
Dempsey et al., 2019	+ + +		+ +	NS	+ +
Gao et al., 2021	+ + +		+ +		
Hong et al., 2014	+ + + +		+ +	NS	
Hong & Chiu, 2016	+ +				
Hou et al., 2017a		+ + +			
Hou et al., 2017b			+ +	-	
Hou et al., 2019			+ +		
Jasso-Medrano & Lopez-Rosales, 2018	+ + +		+ + + , –^a^		
Kircaburun & Griffiths, 2018				–	
Kircaburun et al., 2020a		+ +			
Kircaburun et al., 2020b			+ +	–	
Marino et al., 2016		NS			
Punyanunt-Carter et al., 2018			+ +		
Raza et al., 2020		+ +			
Satici & Uysal, 2015				–	
Sayeed et al., 2020	+ +		+ +		
Shan et al., 2021				–	
Sheldon et al., 2021		+ + , NS^a^			+ + +
Süral et al., 2019		+ +		–-	
Uysal, 2015				–	
Varchetta et al., 2020	+ +				+ + + +
Xie & Karan, 2019	+ + + +		+ +		

*n*: sample size (no. of studies); + : positive association (risk factor); –: negative association (protective factor). Effect size: ±  = very small (VS); +  ±—= small (S); +  +  ± – = medium (M); +  +  +  ± –- = large (L). Interpretation: R^2^: VS > 0 to < 0.1, S ≥ 0.1 to < 0.3, M ≥ 0.3 to < 0.5 and L ≥ 0.5 (Cohen, 2013; Ferguson, 2016); OR: VS > 0 to < 1.5, S ≥ 1.5 to < 2, M ≥ 2 to < 3 and L ≥ 3 (Sullivan & Feinn, 2012); R2: VS > 0 to < 0,02, S ≥ 0,02 to < 0.13, M ≥ 0.13 to < 0.26 and L ≥ 0.26 (Dominguez-Lara, 2017). NS = not significant association

^a^ More than one effect size corresponding to more than one variable as a measure of potential predictor

##### Internet use patterns

*Social media use (time, frequency).*Out of 9 studies, all reported that the use of social media was a potential risk factor for PSMU (Chung et al., 2019; Dempsey et al., 2019; Gao et al., 2021; Hong & Chiu, 2016; Hong et al., 2014; Jasso-Medrano & Lopez-Rosales, 2018; Sayeed et al., 2020; Varchetta et al., 2020; Xie & Karan, 2019). Four reported a small effect size (44.4%), three moderate (33.3%) and two a large effect size (22.2%).

*Social use.* Out of 6 studies, 5 reported that social use of social media was a risk factor for PSMU (Hou et al., 2017a; Kircaburun et al., 2020a; Raza et al., 2020; Sheldon et al., 2021; Süral et al., 2019). Sheldon et al., 2021 found that social activity had a significant effect on Snapchat addiction, with small effect size, but not on Facebook and Instagram addiction. Of the remaining studies that found a significant effect, three reported a small effect size (60%) and one a moderate effect size (20%).

##### Psychological variables

*Negative affect (depression, anxiety, social media communication apprehension, rumination).* Out of 10 studies, all reported negative affectivity as a potential risk factor for PSMU (Dempsey et al., 2019; Gao et al., 2021; Hong et al., 2014; Hou et al., 2019; Jasso-Medrano & Lopez-Rosales, 2018; Kircaburun et al., 2020b; Punyanunt-Carter et al., 2018; Sayeed et al., 2020; Xie & Karan, 2019). Nine showed a small effect size (90%) and one a mixed one (10%).

Specifically, the studies found depression (Gao et al., 2021; Hong et al., 2014; Hou et al., 2019; Jasso-Medrano & Lopez-Rosales, 2018; Kircaburun et al., 2020b; Sayeed et al., 2020), anxiety (Gao et al., 2021; Hou et al., 2019; Xie & Karan, 2019), perceived stress (Hou et al., 2017b), Social media Communication Apprehension (Punyanunt-Carter et al., 2018) and rumination (Dempsey et al., 2019) to be risk factors. In addition, Jasso-Medrano and Lopez-Rosales (2018) found a significant and negative effect of suicidal ideation on PSMU.

*Well-being (flourishing, life satisfaction, social safeness, relationship satisfaction, trait emotional intelligence, self-confidence, self-esteem, vitality, self-liking, psychological capital, psychological resilience).* Out of 11 studies, nine reported that well-being was a potential protective factor against PSMU (Aladwani & Almarzouq, 2016; Demircioğlu & Göncu Köse, 2021; Hou et al., 2017b; Kircaburun & Griffiths, 2018; Kircaburun et al., 2020b; Satici & Uysal, 2015; Shan et al., 2021; Süral et al., 2019; Uysal, 2015). One showed a very small effect size (11.1%), seven showed a small one (77.8%) and one was moderate (11.1%).

Specifically, the studies found the following to be protective factors: flourishing (Satici & Uysal, 2015; Uysal, 2015), self-esteem (Aladwani & Almarzouq, 2016; Demircioğlu & Göncu Köse, 2021), life satisfaction (Satici & Uysal, 2015), social safeness (Uysal, 2015), relationship satisfaction (Demircioğlu & Göncu Köse, 2021), ‘Trait emotional intelligence (TEI)’ (Süral et al., 2019), self-confidence (Kircaburun et al., 2020b), subjective vitality (Satici & Uysal, 2015), self-liking (Kircaburun & Griffiths, 2018), psychological capital (Shan et al., 2021) and psychological resilience (Hou et al., 2017b).

*Fear of Missing Out (FOMO).* Out of 4 studies, all reported FOMO as a potential risk factor for PSMU (Casale et al., 2018; Dempsey et al., 2019; Sheldon et al., 2021; Varchetta et al., 2020). One showed a small effect size (25%), two moderate (50%) and one a large effect size (25%).

### Internet gaming disorder

A total of nine studies have analysed the predictive factors for IGD in university students.

#### Description of studies

The design was longitudinal in four studies (44.4%) (Dang et al., 2019; Yang et al., 2021a, 2021b; Yuan et al., 2021; Zhang et al., 2019) and in five it was cross-sectional (55.6%). 77.8% are from Asia (*n* = 7), one from the USA (Mills & Allen, 2020) and another from Mexico (Borges et al., 2019). 22.2% of the studies were published between 2016—2017, and 77.8% from 2021. The samples ranged from 179 (Kim & Kim, 2017) to 7022 students (Borges et al., 2019), with 88.9% below 1000 and a mean of 1131.9 (SD = 149.5).

The terms ‘Internet gaming disorder’ (*n* = 7, 77.8%), ‘online game addiction’ (*n* = 1, 11.1%) and ‘excessive online game usage’ (*n* = 1, 11.1%) were used.

Eight assessment instruments were identified (see Table 4). High scores indicated a higher degree of IGD. Two studies used the DSM-5 scale (APA, 2013) with a range of 0 to 9 and scores ranging from 1.44 (SD = 1.97) (Zhang et al., 2019) to 1.45 (SD = 1.97) (Dang et al., 2019).

Two studies (22.2%) reported the prevalence. The study by Li et al. (2016), which used the Chinese version of the Online Game Cognitive Addiction Scale (OGCAS; Li et al., 2008), with a range of 16—80 and using a cut-off point of ≥ 32 (plus a score in the ≥ 5 CIA), reported a prevalence of 4.7%. The study by Borges et al., (2019), using an instrument based on the nine symptoms described in DSM-5 and formulated by Petry et al. (2015), with a range of 0—23 and using a cut-off point of ≥ 5, reported a prevalence of 5.2%.

#### Predictive factors

From the nine studies with adequate quality, a potential predictive factor for IGD was extracted, in the category of psychological variables (see Table 7).

**Table 7:** Internet gaming disorder predictive factors

	Psychological variables
	Negative affectivity (depression, avoidant coping styles)
*n*	3
Nº de sujetos	1,278
Dang et al., 2019	+ +
Li et al., 2016	+ +
Yuan et al., 2021	+ + +

*n*: sample size (no. of studies); + : positive association (risk factor); –: negative association (protective factor). Effect size: ±  = very small (VS); +  ±—= small (S); +  +  ± – = medium (M); +  +  +  ± –- = large (L). Interpretation: ®: VS > 0 to < 0.1, S ≥ 0.1 to < 0.3, M ≥ 0.3 to < 0.5 and L ≥ 0.5 (Cohen, 1988; Ferguson, 2009); OR: VS > 0 to < 1.5, S ≥ 1.5 to < 2, M ≥ 2 to < 3 and L ≥ 3 (Sullivan & Feinn, 2012); R^2^: VS > 0 to < 0,02, S ≥ 0,02 to < 0.13, M ≥ 0.13 to < 0.26 and L ≥ 0.26 (Dominguez-Lara, 2017). NS = not significant association

##### Psychological variables

*Negative affectivity (depression, avoidant coping styles).* Out of three studies, all reported negative affectivity as a potential risk factor for IGD (Dang et al., 2019; Li et al., 2016; Yuan et al., 2021). Two showed a small effect size (66.6%) and one a moderate one (33.3%). Specifically, two studies established that depression was a risk factor (Dang et al., 2019; Yuan et al., 2021) while one established avoidant coping style as a risk factor (Li et al., 2016).

### Problematic pornography use

A total of two studies have analysed the predictive factors for problematic internet pornography use (PIPU) in university students.

#### Description of studies

The design was longitudinal in the study by Grubbs et al. (2018) and cross-sectional in that of Chen et al. (2018). Both were published in 2018. The samples were 808 (Chen et al., 2018) and 1507 (Grubbs et al., 2018).

The terms ‘problematic pornography use’ (Chen et al., 2018) and ‘perceived addiction to Internet pornography’ (Grubbs et al., 2018) were used.

Regarding the assessment instruments (see Table 4), the study by Chen et al. (2018) used the Problematic Pornography Use Scale (Kor et al., 2014) reporting an average score of 7.13 (SD = 8.48) in a score range between 0 and 60 (the higher the score, the higher the degree of problematic use). The study by Grubbs et al. (2018) used the Cyber Pornography Use Inventory‐9 (CPUI‐9) (Short et al., 2012) reporting an average score of 1.7 (0.9) in a score range of 1 to 7 (higher score, higher grade of problematic use). Neither study reported on the prevalence.

#### Predictive factors

Due to a lack of studies, no predictive factors were extracted from PIPU.

**Fig. 2:**
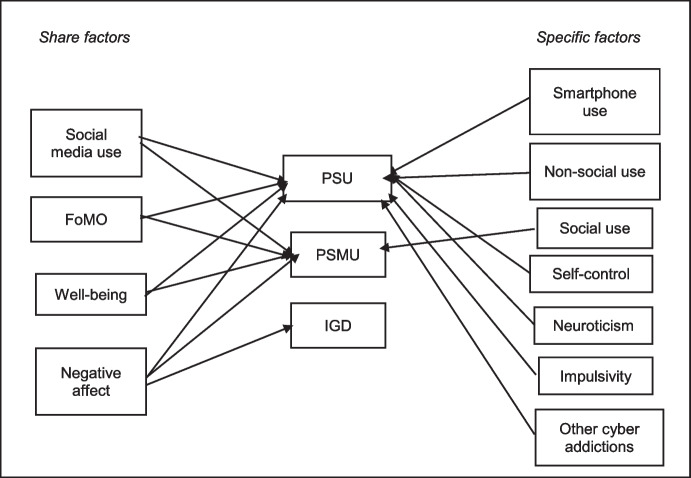
Predictive factors. Note. PSU: Problematic Smartphone use; PSMU: Problematic social media use; IGD: Internet gaming disorder; FoMO: Fear pf Missing Out

Common and specific factors for problematic Internet and smartphone uses are summarised in Fig. 2.

## Discussion and conclusions

Based on previous studies affirming that university students are a population at risk from PIU (Anderson et al., 2017; Ferrante & Venuleo, 2021; Kuss et al., 2014), that PSU and IUP as behaviours overlap in many ways (Carbonell et al., 2018) and that various forms of PIU, including widespread PIU and problematic use of the Internet associated with specific activities (Billieux, 2012; Davis, 2001), the interest in this systematic review has been to complete the study of predictive factors for generalized PIU in this population (Sanchez-Fernandez et al., 2022), focusing in this case on PSU and the specific problem online behaviours that constitute PIU.

As methodological aspects of this systematic review, we can highlight, in the first place, the analysis of international studies with a cross-cultural approach. In addition, the date of bibliographic search, since 2013, is relevant, as this year coincides not only with the date of publication of DSM-5 (APA, 2013), where IGD is acknowledged for the first time, but also with the expansion of smartphones, which make it easier to connect to Internet (Carbonell et al., 2018). Regarding the search strategy, the multiple terms used in the literature to refer to PSU and the specific behaviours within PIU, have been taken into account, which makes it possible to analyse a wide range of studies on these constructs. Finally, with regard to the exhaustive study of predictors, strict criteria have been used in terms of the number of studies that support it, and this furnishes our review with scientific evidence.

With respect to objective 1, to become familiar with the terminology used to refer to PSU and the specific behaviours within PIU, a wide variety of concepts have been found and divided into four groups and subsequently used for the analysis of predictive factors (i.e., PSU, PSMU, IGD and PIPU). Based on the number of articles in each group, research on cyber addictions in university students in recent years has been more focused on the first two (PSU and PSMU). However, no consensus has been found regarding the use of different terms within each group even though there may be differences between them. For example, according to some authors (Kaplan & Haenlein, 2010; Kuss & Griffiths, 2017) ‘social networking’ and ‘social media’ are different concepts even though they are often used interchangeably in literature. The use of ‘social media’ refers to producing and sharing content online, including collaborative projects (e.g., Wikipedia), blogs or microblogs (e.g., Wordpress), content communities (e.g., Flickr), social networking sites (e.g., Instagram) and virtual worlds (e.g., Second Life); while the use of social networks refers to the connection of users (Hamm et al., 2013). This lack of nosological precision has also been reported in other problematic online behaviours such as generalized PIU or PSU (Carbonell et al. (2018), where problematic use and addiction, despite having been established at source as different levels of severity within the same continuum (Young, 1998a; Zhou et al., 2018), are used in research as synonyms.

On the other hand, it should be said that in the case of the PSMU, some studies have analysed the problematic use of specific applications such as “Facebook”, “Instagram” or “WeChat”. However, it is considered advisable to study PSMU in a general way by extending problematic use to a wide range of activities that can take place on social networks, with problematic use of specific social networks such as Facebook being just one example of the PSMU (Kuss & Griffiths, 2017).

In relation to Objective 2, to review the instruments used to assess PSU and specific behaviours of PIU, the instruments that have been highlighted by their frequency are Smartphone Addiction Scale (SAS) (Kwon et al., 2013), in its various forms, in the case of assessing PSU, and the different Bergen scales in assessment of PSMU. However, many different instruments have been found in each of the problematic behaviour groups. Although most are specific to each behaviour, other instruments used are specific to generalized assessment of PIU, such as the IAT (Young, 1998a) in the assessment of PSMU (Hong & Chiu, 2016; Hong et al., 2014; Jaradat & Atyeh, 2017; Kircaburun & Griffiths, 2018) and IGD (Yang et al., 2021a, 2021b). This may be related to the conceptualisation of PIU as an umbrella term that encompasses a number of problematic behaviours on the Internet (Griffiths, 1998, 1999). This heterogeneity and lack of consistency has been confirmed by previous studies that establish the need to develop more advanced assessment instruments in the field of cyber addictions that improve their psychometric properties and allow for a reliable diagnosis (Bányai et al., 2017; Chen & Jiang, 2020; Darvesh et al., 2020; Harris et al., 2020; King et al., 2020; Kuss et al., 2014; Petry et al., 2014; Pontes & Griffiths, 2014; Stevens et al., 2021). A review of Busch and McCarthy (2021) reveals a lack of research to test the functioning of the various PSU measures. In addition, Ryding and Kuss (2020) argue that self-reporting measures are inadequate as we are dealing with unconscious behaviours that are difficult to estimate retrospectively, and therefore propose objective passive monitoring in smartphone research.

In fact, the variety of terminology and assessment tools has affected Objective 3 of this review, which is to analyse the prevalence of PSU and specific PIU behaviours among university students. In the case of PSU, prevalence rates have ranged from 8.99% (Jiang & Shi, 2016), using the Problematic Use of Mobile Phones Scale (PUMPS) (Merlo et al., 2013), to 52.9% (Lian & You, 2017) using the Mobile Phone Addiction Scale Index (MPAI) (Leung, 2008). In the case of PSMU, prevalence rates varied from 14.8% (8.2% ‘‘Excessive use” and 6.6% “serious excessive use”) (Hou et al., 2017a) using the ‘Excessive WeChat Use scale’, to 70% (62.1% with alert levels and 7.9% with addiction) (Jaradat & Atyeh, 2017), using the Internet Addiction Test (IAT) (Young, 1998a). Regarding IGD, prevalence rates ranged from 4.7% (Li et al., 2016), using the Online Game Cognitive Addiction Scale (OGCAS; Li et al., 2008), to 5.2% (Borges et al., 2019), using the Petry et al. instrument. (2015).

However, variability in prevalence rates may be due to other factors. In this respect, studies with the same instrument and the same cut-off point (≥ 31 in men and ≥ 33 in women in the Smartphone Addiction Scale Short Version), prevalence rates ranged from 27.92% (Yuchang et al., 2017) to 48.6% (Gündoğmuş et al., 2021). So, these discrepancies could be explained by socio-cultural differences among users at university (Bányai et al., 2017; Lopez-Fernandez et al., 2017).

In spite of the variability found, the prevalence has generally been higher in PSU and PSMU than in IGD, which may be due to the fact that the first two constructs include a greater number of problematic behaviours. However, these results should be interpreted with caution as the percentage of studies that reported prevalence rates was very low. This in turn could be due to a current conceptualisation of PIU based on a dynamic and procedural view, according to which we would be dealing with differences between levels of severity within a continuum from "normal" to pathological (Ferrante & Venuleo, 2021).

With regard to Objective 4, to study the risk and protective factors associated with PSU and specific problem behaviours online in university students, 10 associated with PSU, four associated with PSMU and one associated with IGD were found, categorised into two types of factors: patterns of use and psychological variables. Following the study by Billieux (2012) these can in turn be categorised as being common to different problematic behaviours or specific to each one of them.

Beginning with predictors common to more than one problematic behaviour, in terms of usage patterns, evidence has been found to affirm that social media use increases the risk of PSU and PSMU in university students (Abbasi et al., 2021; Chung et al., 2019; Coban & Gundogmus, 2019; Dempsey et al., 2019; Gao et al., 2021; Hong & Chiu, 2016; Hong et al., 2014; Jasso-Medrano & Lopez-Rosales, 2018; Koç & Turan, 2021; Lin & Chiang, 2017; Rozgonjuk et al., 2018; Salehan & Negahban, 2013; Sayeed et al., 2020; Varchetta et al., 2020; Xie & Karan, 2019). This is in line with studies which affirm that, among PIU-specific behaviours, using social media has a higher risk of becoming problematic (Carbonell et al., 2018). This result is important because academic use of social media has increased in recent years (León-Gómez et al., 2021; Seaman & Tinti-Kane, 2013), making it necessary that the introduction of social networks in the classroom is accompanied by training in healthy use of social media so as not to increase the risk of problematic behaviour among students.

Regarding psychological variables, negative affectivity was a risk factor common to PSU, PSMU and IGD (Alavi et al., 2020; Canale et al., 2021; Choi et al., 2015; Cui et al., 2021; Dang et al., 2019; Dempsey et al., 2019; Elhai et al., 2018a; Elhai et al., 2020c; Enez Darcin et al., 2016; Forster et al., 2021; Gao et al., 2021; He et al., 2020; Hong et al., 2014; Hou et al., 2021; Hou et al., 2019; Jasso-Medrano & Lopez-Rosales, 2018; Khoury et al., 2019; Kim & Koh, 2018; Kim et al., 2017; Kim et al., 2019; Kircaburun et al., 2020b; Kuang-Tsan & Fu-Yuan, 2017; Kuru & Celenk, 2021; Li et al., 2016; Liu et al., 2020; Liu et al., 2021; Long et al., 2016; Matar Boumosleh & Jaalouk, 2017; Punyanunt-Carter et al., 2018; Sayeed et al., 2020; Xiao et al., 2021; Xie & Karan, 2019; Yang et al., 2020a, 2020b; You et al., 2019; Yuan et al., 2021; Zhang et al., 2020a). In fact, the previous review had already found a risk factor for generalized PIU (Sanchez-Fernandez et al., 2022). This outcome is consistent with model of compensatory internet use aetiological models that suggest that these problematic behaviours may reflect maladaptive coping deployed to regulate negative moods or cope with affective disorders (Kardefelt-Winther, 2014; Kardefelt-Winther et al., 2017), and with cognitive behavioural model of pathological Internet use (Davis, 2001). In line with this, well-being was found to be a protective factor for PSU and PSMU (Aladwani & Almarzouq, 2016; Demircioğlu & Göncu Köse, 2021; Elhai et al., 2018b; Hou et al., 2017b; Jiang & Shi, 2016; Kim & Koh, 2018; Kircaburun & Griffiths, 2018; Kircaburun et al., 2020b; Koç & Turan, 2021; Lian & You, 2017; Lian, 2018; Satici & Uysal, 2015; Shan et al., 2021; Sun et al., 2021; Süral et al., 2019; Uysal, 2015; Yuchang et al., 2017; Zhang et al., 2020). However, there may be two-way relationships between negative affectivity and cyber addictions. Thus, the updated person-affect-cognition-execution interaction model (Brand et al., 2019) states that in the early stages of problematic behaviour, relief in negative affective responses would lead to positive reinforcement which in turn would lead to the establishment of problematic behaviour. As the process progresses and control over the use of specific Internet activities decreases, negative affectivity may be exacerbated by repeated use of the chosen online sites/applications, and problematic behaviour is maintained due to compensatory effects.

This is in line with Busch and McCarthy (2021) who find in their review that emotional health problems are a background to, but also a consequence of PSU, suggesting the need to define and determine how these variables relate. On the other hand, the relationships between cyberaddictions and negative affectivity are not static and can be affected by situational circumstances and traumatic events (Chen et al., 2022).

On the other hand, the Fear of Missing Out (FOMO) – defined as anxiety arising from the belief that others may be having rewarding social experiences which you are not included in (Przybylski et al., 2013) – has been found to be a risk factor for PSU and PSMU (Casale et al., 2018; Dempsey et al., 2019; Elhai et al., 2018b, 2020a, 2020b, 2020c; Handa & Ahuja, 2020; LIN et al., 2021; Sheldon et al., 2021; Varchetta et al., 2020; Wolniewicz et al., 2020; Yuan et al., 2021). Kuss and Griffiths (2017) state that FOMO may be part of social media addiction.

Based on these findings, the introduction in universities of actions aimed at promoting appropriate stress coping strategies and, in general, mental health for the prevention of online problem behaviours is proposed. School-based social and emotional learning (SEL) programs are proposed in the literature (Barry et al., 2017; Dowling & Barry, 2020), which are based on the integration of actions aimed at promoting mental health in teaching practices, and are proven to be effective. This would be in line with the current lines of treatment according to which it would be about, on the one hand, having an impact on emotional health and treating concurrent disorders, such as depression or anxiety (Király & Demetrovics, 2021).

Following the specific predictive factors, in terms of patterns of use, it has been found that the use of the smartphone, in time and frequency, is a risk factor for PSU (Alosaimi et al., 2016; Elhai et al., 2018a, 2018c, 2020b; Erdem & Uzun, 2020; Gökçearslan et al., 2016; Hong et al., 2021; Lin et al., 2021; Wolniewicz et al., 2020). In line with this, the amount of time spent online is a predictor of PIU (Sanchez-Fernandez et al., 2022). In fact, in cognitive-behavioural therapy for problematic Internet use, one of the techniques used is usage monitoring with the goal of reducing the amount of time spent online to a degree that no longer interferes with the client's healthy functioning (Király & Demetrovics, 2021). In the university setting, one way to act on this risk factor would be to promote curricular and extracurricular activities that do not involve smartphone use so that university students do not spend so much time online. However, it must be noted that the studies reviewed have considered the variable ‘time of use’ without differentiating the activities carried out on the network. Huang's meta-analysis (2010) establishes that the effect of internet usage time on PIU is moderated by specific activities (e.g., social vs. non-social). It is therefore recommended that these psychometric limitations be solved by studying the effect of time spent on PSU, distinguishing between time spent on different functions (such as academic, work or entertainment).

In addition, a positive effect of process use—defined as smartphone use involving non-social motivations such as news consumption, entertainment, and relaxation—has also been found in PSU (Elhai et al., 2020a; Rozgonjuk & Elhai, 2021; Rozgonjuk et al., 2019). On the other hand, social use -creating and maintaining relationships- has been found to be a risk factor of PSMU (Hou et al., 2017a; Kircaburun et al., 2020a; Raza et al., 2020; Sheldon et al., 2021; Süral et al., 2019). For this reason, it would be advisable for university institutions to favor alternative forms of face-to-face entertainment and the establishment of social relationships among students.

With respect to psychological variables that specifically predict PSU, impulsivity should be noted (Canale et al., 2021; Khoury et al., 2019; Roberts & Pirog III, 2013; Roberts et al., 2015). It has also been confirmed as a risk factor for generalized PIU (Sanchez-Fernandez et al., 2022). This can be explained by aetiological models that argue that these problematic behaviours may reflect impulse control disorders (Kardefelt-Winther et al., 2017; Young, 1998a). In addition, the PSU model developed by Pivetta et al. (2019), suggests that attention impulsivity predicts addictive and antisocial use of the mobile phone. In the same vein, self-control/self-regulation has been found to be a protective factor for PSU (Cebi et al., 2019; Gökçearslan et al., 2016; Jiang & Shi, 2016; Pourrazavi et al., 2014; Yang et al., 2019, 2020a, 2020b; Zhang et al., 2021), which could be explained by the Larose model of self-regulation (2003), so that people with good levels of self-regulation would be able to activate self-conscious processes that would allow them to judge, monitor and adjust their behaviour online. In fact, Billieux (2012) describes an integrative model in the origin of PSU that includes the impulsive pathway among the different ones. This pathway describes those individuals whose mobile use is motivated by poor self-control and/or poor regulation of emotions. Therefore, preventive strategies that promote self-control and emotional regulation among students could be implemented. For example, mindfulness-based stress reduction (MBSR) intervention has positive effects on the mental health of college students (Canby et al., 2015).

Also, neuroticism/emotional stability is a personality trait that predicts PSU (De Pasquale et al., 2019; Erdem & Uzun, 2020; Liu et al., 2020; Roberts et al., 2015; Takao, 2014). This finding is consistent with the Pivetta et al. model (2019), which establishes a positive relationship between neuroticism and addictive smartphone use. This can be understood through the pathway of excessive reaffirmation (Billieux et al., 2015a) according to which inappropriate use of the mobile phone would be explained by the perceived need to maintain interpersonal relationships and to be constantly encouraged by others.

Finally, other cyber addictions have been found to be a risk factor for PSU (Choi et al., 2015; Khoury et al., 2019; Yuan et al., 2021), which could be explained by another of the pathways of the Billieux (2012) model: the pathway of cyber addiction. Smartphones allow access to the internet and various online activities and so people who make dysfunctional use of the internet or some of these activities would be more susceptible to misuse their smartphone. In fact, this result is in line with the predictors common to the different behaviours found in this review and in the previous review (Sanchez-Fernandez et al., 2022). As a result, there could be mechanisms that would explain the entire spectrum of cyber addictions such as negative affectivity, and it would be very important to promote it from universities.

### Practical implications

This systematic review allow to achieve greater knowledge about PSU and about specific problematic uses of Internet in university students and their predictive factors. The findings may be useful in the development of preventive educational strategies that, implemented from early stages, such as in primary and secondary education, and continuously as lifelong learning, could reduce the occurrence of these online problem behaviours in the university stage. In this way, we are not only contributing to the research needs in this area proposed by the WHO (2015) but also to current European educational policies that aim to support a sustainable and effective adaptation of education and training systems to the digital age (European Commission, 2020). In this way, the ultimate aim is for students to make use of the internet to enable them to be active citizens in the present knowledge society and, at the same time, to minimize the negative repercussions of the network on physical, psychological and social health.

### Limitations and future research

Regarding the limitations of the studies included in this review, the number of studies found that met the inclusion criteria and focused on IGD and PIPU was small, and there was a lack of online gambling studies, which has made it impossible to obtain predictors of these behaviours. In addition, it should be noted that most of the studies included have not met the quality criterion with respect to control of extraneous variables. This means the results obtained have to be treated with caution.

On the other hand, with respect to the limitations of the review itself, in the first place, only the direct effects of the potential predictor variables of problem behaviours have been studied, without taking into account variables that had an indirect effect on the behaviours studied. In future studies, we recommend paying attention to the variables that indirectly influence these behaviours. Secondly, because most of the studies included had a cross-sectional design, with the data extracted it is impossible to determine the causal nature of the predictive factors studied. Thirdly, a very high percentage of studies were conducted in Asia, which may skew the results when generalising for other regions. For this reason, the proposal is to replicate this study in other continents so that the results can be made specific and compared. And fourthly, although the most relevant databases have been used in the topic studied, there could be an information bias that might make it necessary to extend the study using databases not taken into account in this paper.

This study continues the line of research consisting in the systematization of common and specific predictive factors of the different problematic online behaviours, providing a new approach focused on the university population. Based on the findings of this review, further research is needed on predictors of problematic online behaviour such as IGD, PIPU and problem online gambling in this population. Also, future research should use longitudinal designs to establish causal relationships between predictive variables and PSU/sPIU. Regarding recommendations for future prevention programs, these should target to the development of adaptive coping strategies that allow an adequate response to negative emotional states, working in parallel with usage patterns that increase the risk of inappropriate internet use.

## Conclusions

In summary, this systematic review makes it possible, on the one hand, to reaffirm the need to continue moving forward with the conceptualisation and assessment of PSU and specific PIU in order to achieve consistent diagnostic criteria that, in turn, make it possible to establish the prevalence of these problems in the population in general and in university students in particular. On the other hand, with regard to the predictors reported, our results support the updated version of the I-PACE model (Brand et al., 2019), with the result that the patterns of use and the psychological variables observed increase the prior vulnerability of specific online problematic behaviours by acting as predisposing factors. These behaviours, by interacting with affective and cognitive responses to stimuli, deficits in executive functioning, decision-making behaviour that leads to use of certain applications/Internet sites and the consequences of use of applications/Internet sites, would lead to the development and perpetuation of problem behaviour online. Our findings have made it possible to make progress in the investigation of shared and specific predictive factors of problem behaviours online, thus allowing the formulation of preventive strategies aimed at each one of them.

## Data Availability

All data generated or analysed during this study are included in this published article.

## Appendix

PRISMA 2009 Checklist

*From:* Moher D, Liberati A, Tetzlaff J, Altman DG, The PRISMA Group (2009). Preferred Reporting Items for Systematic Reviews and Meta-Analyses: The PRISMA Statement. PLoS Med 6(7): e1000097. 10.1371/journal.pmed1000097.

**Table:**

Section/topic	#	Checklist item	Reported on page #
**TITLE**			
Title	1	Identify the report as a systematic review, meta-analysis, or both	1
**ABSTRACT**			
Structured summary	2	Provide a structured summary including, as applicable: background; objectives; data sources; study eligibility criteria, participants, and interventions; study appraisal and synthesis methods; results; limitations; conclusions and implications of key findings; systematic review registration number	1
**INTRODUCTION**			
Rationale	3	Describe the rationale for the review in the context of what is already known	2 – 6
Objectives	4	Provide an explicit statement of questions being addressed with reference to participants, interventions, comparisons, outcomes, and study design (PICOS)	6
**METHODS**			
Protocol and registration	5	Indicate if a review protocol exists, if and where it can be accessed (e.g., Web address), and, if available, provide registration information including registration number	1
Eligibility criteria	6	Specify study characteristics (e.g., PICOS, length of follow-up) and report characteristics (e.g., years considered, language, publication status) used as criteria for eligibility, giving rationale	6
Information sources	7	Describe all information sources (e.g., databases with dates of coverage, contact with study authors to identify additional studies) in the search and date last searched	6
Search	8	Present full electronic search strategy for at least one database, including any limits used, such that it could be repeated	6
Study selection	9	State the process for selecting studies (i.e., screening, eligibility, included in systematic review, and, if applicable, included in the meta-analysis)	7
Data collection process	10	Describe method of data extraction from reports (e.g., piloted forms, independently, in duplicate) and any processes for obtaining and confirming data from investigators	8
Data items	11	List and define all variables for which data were sought (e.g., PICOS, funding sources) and any assumptions and simplifications made	8
Risk of bias in individual studies	12	Describe methods used for assessing risk of bias of individual studies (including specification of whether this was done at the study or outcome level), and how this information is to be used in any data synthesis	8
Summary measures	13	State the principal summary measures (e.g., risk ratio, difference in means)	8
Synthesis of results	14	Describe the methods of handling data and combining results of studies, if done, including measures of consistency (e.g., I^2^) for each meta-analysis	8
Risk of bias across studies	15	Specify any assessment of risk of bias that may affect the cumulative evidence (e.g., publication bias, selective reporting within studies)	8
Additional analyses	16	Describe methods of additional analyses (e.g., sensitivity or subgroup analyses, meta-regression), if done, indicating which were pre-specified	8
**RESULTS**			
Study selection	17	Give numbers of studies screened, assessed for eligibility, and included in the review, with reasons for exclusions at each stage, ideally with a flow diagram	101
Study characteristics	18	For each study, present characteristics for which data were extracted (e.g., study size, PICOS, follow-up period) and provide the citations	58—84
Risk of bias within studies	19	Present data on risk of bias of each study and, if available, any outcome level assessment (see item 12)	58—84
Results of individual studies	20	For all outcomes considered (benefits or harms), present, for each study: (a) simple summary data for each intervention group (b) effect estimates and confidence intervals, ideally with a forest plot	58—84
Synthesis of results	21	Present results of each meta-analysis done, including confidence intervals and measures of consistency	8—17
Risk of bias across studies	22	Present results of any assessment of risk of bias across studies (see Item 15)	8
Additional analysis	23	Give results of additional analyses, if done (e.g., sensitivity or subgroup analyses, meta-regression [see Item 16])	
**DISCUSSION**			
Summary of evidence	24	Summarize the main findings including the strength of evidence for each main outcome; consider their relevance to key groups (e.g., healthcare providers, users, and policy makers)	17 – 23
Limitations	25	Discuss limitations at study and outcome level (e.g., risk of bias), and at review-level (e.g., incomplete retrieval of identified research, reporting bias)	24
Conclusions	26	Provide a general interpretation of the results in the context of other evidence, and implications for future research	25
**FUNDING**			
Funding	27	Describe sources of funding for the systematic review and other support (e.g., supply of data); role of funders for the systematic review	1
